# Identification of the molecular characteristics associated with microsatellite status of colorectal cancer patients for the clinical application of immunotherapy

**DOI:** 10.3389/fphar.2023.1083449

**Published:** 2023-02-06

**Authors:** Jie Fu, Xiaoxin Jin, Weidong Chen, Zongyao Chen, Peidong Wu, Wang Xiao, Yuhang Liu, Shuangya Deng

**Affiliations:** Department of General Surgery, The Second Xiangya Hospital of Central South University, Changsha, China

**Keywords:** colorectal cancer, microsatellite stability, single-cell, immunotherapy, risk score

## Abstract

**Background:** Mismatch repair-proficient (pMMR) microsatellite stability (MSS) in colorectal cancer (CRC) indicates an unfavorable therapeutic response to immunotherapy with immune checkpoint inhibitors (ICIs). However, the molecular characteristics of CRC patients with pMMR MSS remain largely unknown.

**Methods:** Heterogeneities between mismatch repair-deficient (dMMR) microsatellite instability (MSI) and pMMR MSS CRC patients were investigated at the single-cell level. Next, an MSS-related risk score was constructed by single-sample gene set enrichment analysis (ssGSEA). The differences in immune and functional characteristics between the high- and low-score groups were systematically analyzed.

**Results:** Based on the single-cell RNA (scRNA) atlas, an MSS-specific cancer cell subpopulation was identified. By taking the intersection of the significant differentially expressed genes (DEGs) between different cancer cell subtypes of the single-cell training and validation cohorts, 29 MSS-specific cancer cell marker genes were screened out for the construction of the MSS-related risk score. This risk score signature could efficiently separate pMMR MSS CRC patients into two subtypes with significantly different immune characteristics. The interactions among the different cell types were stronger in the MSS group than in the MSI group, especially for the outgoing signals of the cancer cells. In addition, functional differences between the high- and low-score groups were preliminarily investigated.

**Conclusion:** In this study, we constructed an effective risk model to classify pMMR MSS CRC patients into two completely different groups based on the specific genes identified by single-cell analysis to identify potential CRC patients sensitive to immunotherapy and screen effective synergistic targets.

## Introduction

Colorectal cancer (CRC) is one of the leading causes of cancer-related death worldwide(Sung et al., 2021; Bray and Parkin, 2022). The treatments of CRC have made great progress in recent years, such as targeted therapy based on gene detection and tumor heterogeneity(Adamopoulos et al., 2021; Kotani et al., 2022). However, the prognosis of CRC patients with advance-stage disease, recurrence and metastasis is still unsatisfactory(André et al., 2022; Ciardiello et al., 2022; Topham et al., 2022).

Immunotherapy represented by immune checkpoint inhibitors (ICIs) has shown good curative effects in many malignant tumors, but its application in CRC is still limited to patients with mismatch repair-deficient (dMMR) microsatellite instability (MSI) status(Jin and Sinicrope, 2022; Weng et al., 2022). The lack of immune infiltration and low tumor mutation burden (TMB) are the main reasons for the insensitivity of CRC patients with mismatch repair-proficient (pMMR) microsatellite stability (MSS) status to immunotherapy(Ganesh et al., 2019). However, the genomic features of approximately 85% of CRC patients are pMMR MSS(Boland and Goel, 2010). Thus, it is urgent to clarify the molecular characteristics of pMMR MSS CRC patients to find effective synergistic targets for immunotherapy.

The differences in immune landscapes between dMMR MSI and pMMR MSS CRC patients have been preliminarily explored at the single-cell RNA (scRNA) level(Bao et al., 2020). However, the overall differences between the two groups have not been fully elucidated, and whether the specific molecular features of pMMR MSS patients are associated with insensitivity to immunotherapy remains largely unknown. Several studies have divided pMMR MSS CRC patients into “hot” and “cold” subtypes according to the abundances of immune cell infiltration based on single-cell RNA (scRNA) and bulk RNA sequencing data, and potential synergistic targets of immunotherapy for “hot” tumors with high abundances of immune cells have also been identified(Picco et al., 2021; Ren et al., 2022; Wang et al., 2022). However, there is still a lack of a single-cell level risk score signature to classify pMMR MSS patients into different subgroups and thus judge their sensitivity to immunotherapy.

In this study, the heterogeneities between dMMR MSI and pMMR MSS CRC patients were systematically elucidated by the scRNA atlas. A specific cancer cell cluster was identified in MSS CRC patients. Furthermore, a specific MSS-related gene list was assembled by integrated analysis of the training and validation scRNA cohorts, which were subsequently used for constructing the MSS-related risk score. This MSS-related risk score could efficiently separate CRC patients into two subgroups with completely different immune characteristics in the external transcriptome cohorts. CRC patients in the low-score group were identified as “hot” tumors with high abundances of immune cells and an inhibitory immune microenvironment, which means they are potentially sensitive to immunotherapy.

## Materials and methods

### Data acquisition

Five CRC patients (SMC03-T, SMC06-T, SMC10-T, SMC24-T and KUL01-T) with MSI-H status (MSI group) and seven CRC patients (SMC23-T, SMC25-T, KUL19-T, KUL21-T, KUL28-T, KUL30-T and KUL31-T) with MSS status (MSS group) were used as a single-cell training cohort (GSE132465 and GSE144735)(Lee et al., 2020), while two CRC patients (T_ cac7 and T_ cac12) with MSI-H status and four CRC patients (T_cac1, T_cac2, T_cac4 and T_cac10) with MSS status were used as a single-cell validation cohort in this study (GSE200997)(Khaliq et al., 2022). In detail, the training cohort comprised the MSI group with 9366 high-quality single cells, and the MSS group with 9603 high-quality single cells. The validation cohort comprised the MSI group with 5555 cells, and the MSS group with 5665 cells. All of the scRNA data were downloaded from the Gene Expression Omnibus (GEO) database (https://www.ncbi.nlm.nih.gov/geo/), and the clinical information of these CRC samples is shown in Supplementary Table S1. In addition, transcriptome data in transcripts per million (TPM) of 601 CRC patients (185 patients with MSI status and 416 patients with MSS status) were downloaded from The Cancer Genome Atlas (TCGA) database (https://portal.gdc.cancer.gov/) as a transcriptome training cohort (TCGA-CRC cohort), while the microarray data of the other 527 CRC patients (73 patients with dMMR status and 454 patients with pMMR status) were downloaded from the GEO database (GSE39582) as a transcriptome validation cohort (GEO-CRC cohort)(Marisa et al., 2013).

### ScRNA data analysis

All of the scRNA data were analyzed by the “Seurat” R package in this study(Satija et al., 2015). Specifically, data normalization, screening of highly variable genes (HVGs), removal of interbatch differences and data integration were conducted by the “SCTransform” function. Subsequently, dimension reduction and cluster analyses were conducted by the uniform manifold approximation and projection (UMAP) and “FindClusters” methods respectively. Significantly highly expressed genes in each cell cluster were identified by the “FindAllMarkers” method. After that, all of the cell clusters were annotated to specific cell types by the specific markers as previously reported (Supplementary Table S2)(Lee et al., 2020; Liu et al., 2022). Moreover, significant differentially expressed genes (DEGs) between different cell clusters were identified by the “FindMarkers” method with a min.pct of 0.5 and logfc.threshold of 2. Finally, cell-cell communication analysis among different cell types was conducted by the “CellChat” R package(Jin et al., 2021).

### Calculation of the risk scores of CRC samples

The risk score of each CRC sample was calculated by the single-sample gene set enrichment analysis (ssGSEA) algorithm based on transcriptome data according to the specific single-cell marker genes. CRC patients in the transcriptome training and validation cohorts were divided into high- and low-score groups according to the median value of the risk score. Survival analysis between the two groups was conducted by the “survMisc” and “survminer” R packages.

### Immune analysis and gene set variation analysis (GSVA)

Abundances of 28 immune cells in each CRC sample were calculated by the ssGSEA method according to the specific markers as previously reported (Supplementary Table S3). Correlations between the abundances of immune cells and the expression levels of single-cell marker genes were calculated by the Spearman correlation analysis method. In addition, functional differences between the high- and low-score groups in the transcriptome training and validation cohorts were identified by GSVA(Hänzelmann et al., 2013).

### Drug prediction

CellMiner is a web tool that predicts the sensitivity of target genes to drugs based on the NCI-60 cell line set(Reinhold et al., 2019). In this study, potential drugs that have been approved by the Food and Drug Administration (FDA) targeting these single-cell marker genes were screened by CellMiner.

### Statistical analysis

All of the data were analyzed by R 4.1.0 in this study. Continuous quantitative data between two groups were compared by Student’s t-test or the Wilcoxon test. Correlation analyses were conducted by the Spearman method. The survival data were analyzed by the log-rank test. A *p*-value <0.05 was considered statistically significant.

## Results

### Single-cell atlas of CRC patients with MSI and MSS status

**FIGURE 1:**
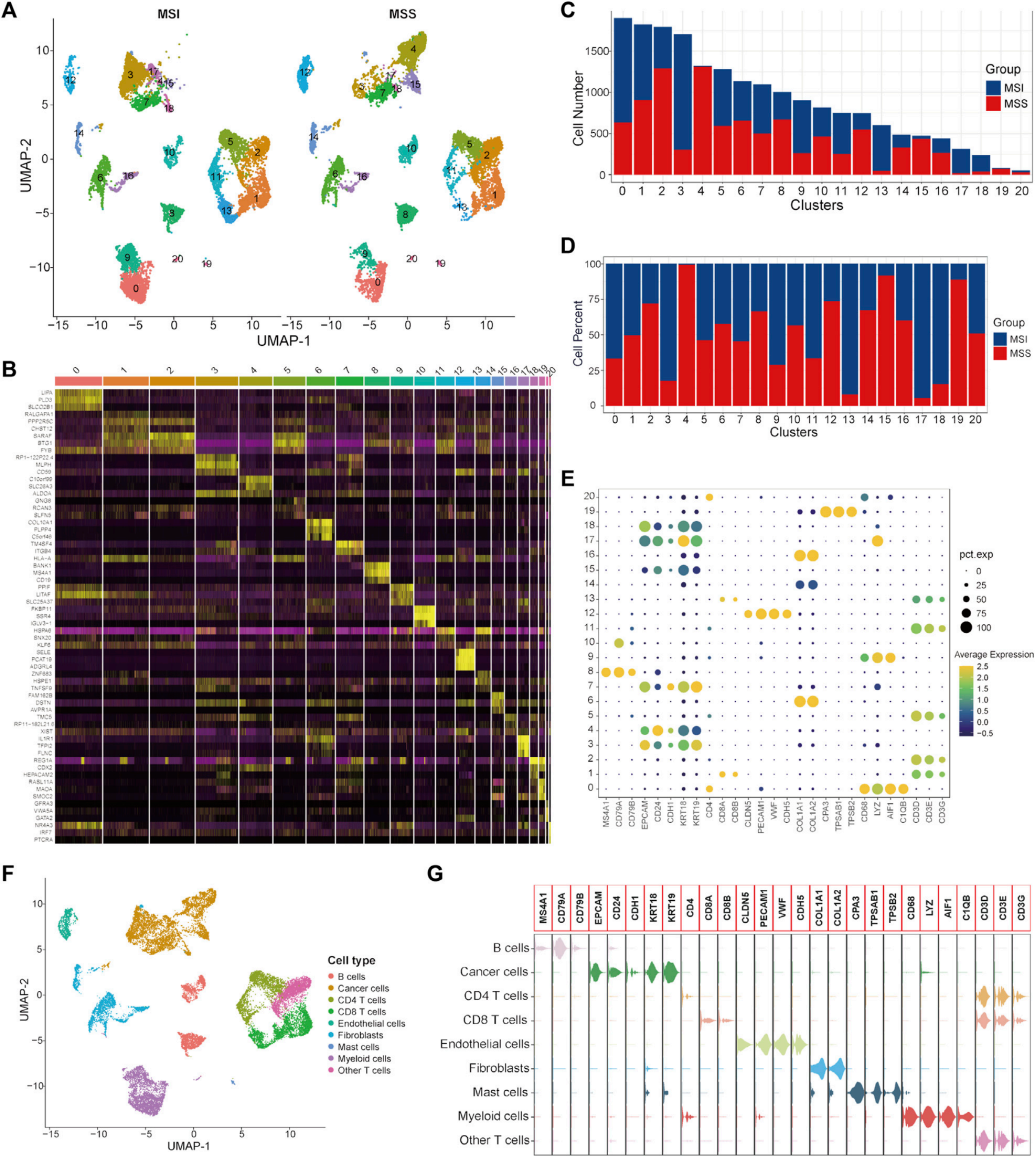
Single-cell atlas of MSI and MSS CRC patients in the scRNA training cohort. **(A)** UMAP and cluster analyses (Clusters 0–20) of 9366 single cells from CRC patients with MSI status and 9603 cells with MSS status. **(B)** The heatmap visualizes the top three significantly highly expressed genes in each cell cluster. **(C)** Cell numbers of MSI and MSS CRC-derived cells in these 21 cell clusters. **(D)** Cell proportions of MSI and MSS CRC-derived cells in these 21 cell clusters. **(E)** Dot plots visualize the specific cell marker genes in each cell cluster. **(F)** UMAP plot of cell annotation results. **(G)** Violin plots of the specific cell marker genes.

A total of 9366 single cells from CRC patients with MSI status and 9603 cells with MSS status were used to construct the single-cell atlas as a training cohort. The expression profiles of the CRC patients with MSI and MSS status, as well as the correlation between the numbers of features and counts of the single cell, are visualized in Supplementary Figures S1A, B. After data normalization, data integration, dimensionality reduction and clustering analyses, the CRC cells were stratified into 21 clusters (Figure 1A and Supplementary Figure S1C), and the top three significantly highly expressed genes in each cluster are shown in Figure 1B. The general differences between the MSI and MSS groups are visualized in Supplementary Figure S1D, while there were no significant batch effects among the different GEO sources, patients and cell cycles (Supplementary Figures S1E–G). The absolute amounts and percentages of these 21 cell clusters from CRC patients with MSI or MSS status are visualized in Figures 1C, D. We note that Clusters 4, 15 and 19 account for a significantly higher proportion in the MSS group (exceeding 75%), while Clusters 3, 13, 17 and 18 account for a significantly higher proportion in the MSI group. To address the functional characteristics of these cell clusters, the cell types were annotated. These 21 cell clusters could be annotated to 9 cell types (B cells, cancer cells, CD4 T cells, CD8 T cells, endothelial cells, fibroblasts, mast cells, myeloid cells and other T cells) according to specific cell markers (Figures 1E, F), and the marker genes for each cell type were visualized by violin plots (Figure 1G). It is noteworthy that Clusters 3, 4, 15, 17 and 18 are all cancer cells, while Cluster 13 is CD8 T cells and Cluster 19 is mast cells. In addition, the proportion of no known cell types was more than 75% in the MSS group, except for mast cells (Supplementary Figures S2A, C).

It has been reported that the enrichment of CD8 T cells in CRC patients with MSI status is an important reason for their relatively higher sensitivity to immunotherapy with ICIs(Sui et al., 2021; Zou et al., 2021). Our analysis results further confirmed this knowledge at the single-cell level. Considering that mast cells only account for a very small proportion of total cells (80/18,969), we mainly focused on cancer cells in this study for subsequent analysis. To further investigate the specific characteristics of the CRC patients with MSS status, DEGs between MSS-specific cancer cell Clusters 4 and 15 and other cancer cell clusters were identified and 153 significant DEGs were screened out (Supplementary Table S4).

### Cell-cell communication analysis

**FIGURE 2:**
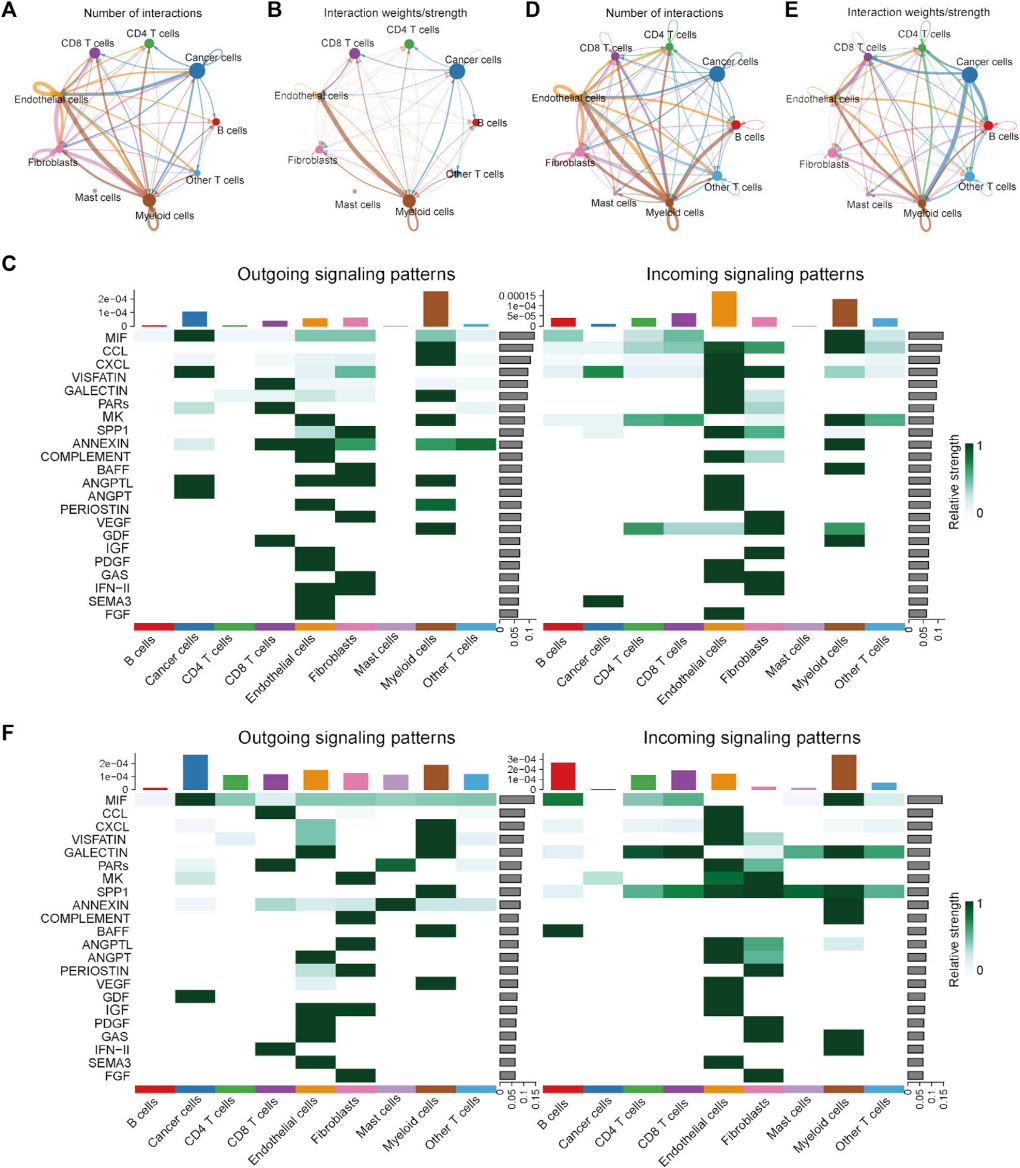
Visualization of cell-cell communication results in the scRNA training cohort. **(A)** Number of interactions among the nine cell types in the MSI group. **(B)** Interaction strength among the 9 cell types in the MSI group. **(C)** The potential incoming and outgoing signaling pathways among the 9 cell types in the MSI group. **(D)** Number of interactions among the 9 cell types in the MSS group. **(E)** Interaction strength among the 9 cell types in the MSS group. **(F)** The potential incoming and outgoing signaling pathways among the 9 cell types in the MSS group.

To investigate the differences in the integrated role among different cell types between the MSI and MSS groups, cell-cell communication analysis was conducted separately. The number and strength of the cell-cell interactions in the MSI group are shown in Figures 2A, B, and the specific intracellular signal information of the MSI group is shown in Figure 2C. We note that the interactions among different cell types were generally weak in the MSI group, and a relatively strong interaction only existed between myeloid cells and endothelial cells (Figures 2B, C). However, the interactions among different cell types were significantly strengthened in the MSS group (Figures 2D, E), and the outgoing signals of various cell types were significantly enhanced, especially for cancer cells (Figure 2F). Moreover, the detailed intercellular signal information of the MSI and MSS groups is visualized in Supplementary Figures S4, S5. These results, combined with the results of the single-cell atlas, revealed the heterogeneities between the MSS and MSI groups, and the potential core roles of specific cancer cell subtypes in MSS CRC patients were also preliminarily elucidated.

### External validation of the specific cancer cell subtypes in CRC patients with MSS status

**FIGURE 3:**
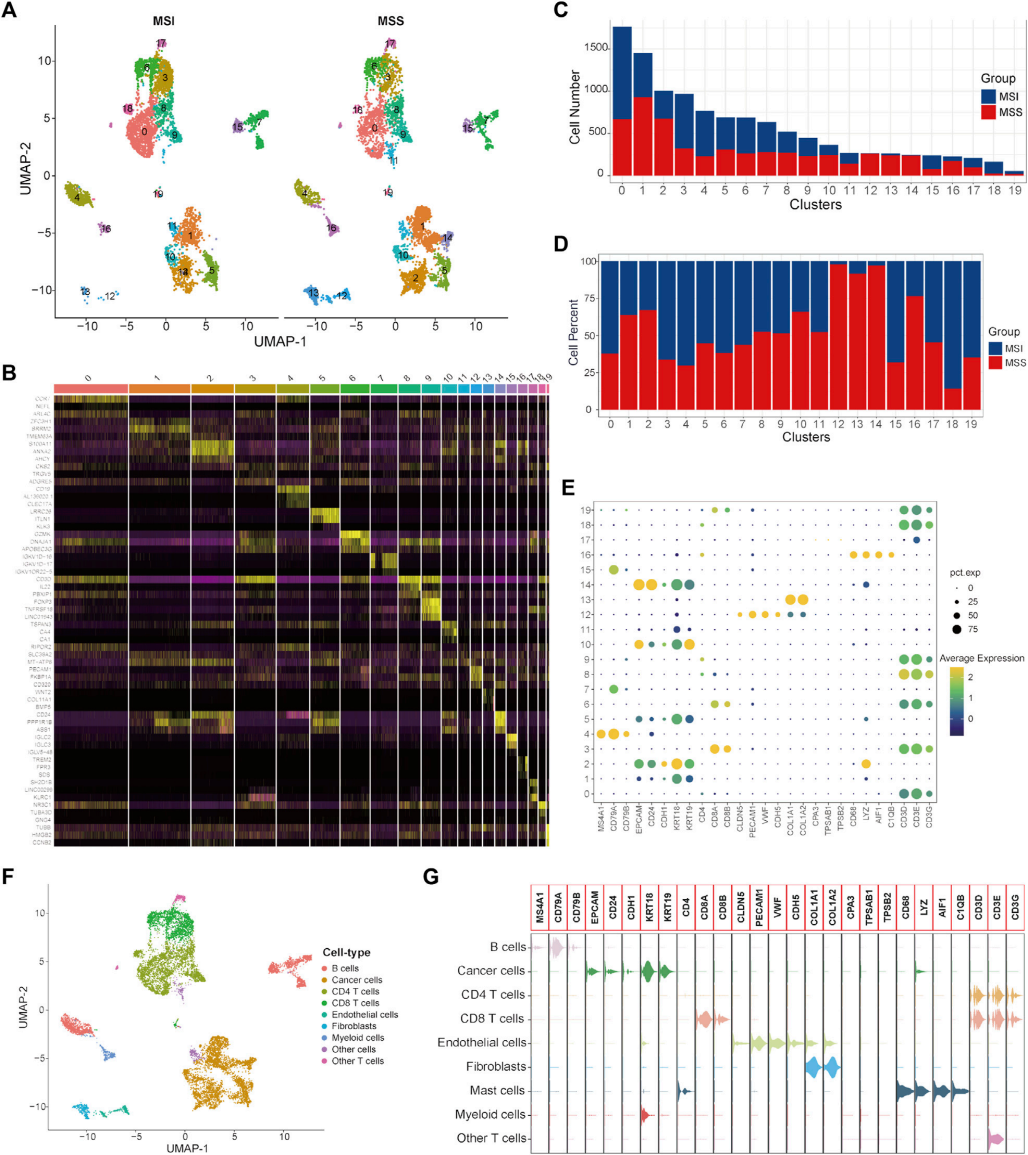
Single-cell atlas of MSI and MSS CRC patients in the scRNA validation cohort. **(A)** UMAP and cluster analyses (Clusters 0–19) of 5555 single cells from CRC patients with MSI status and 5665 cells with MSS status. **(B)** The heatmap visualizes the top three significantly highly expressed genes in each cell cluster. **(C)** Cell numbers of MSI and MSS CRC-derived cells in these 20 cell clusters. **(D)** Cell proportions of MSI and MSS CRC-derived cells in these 20 cell clusters. **(E)** Dot plots visualize the specific cell marker genes in each cell cluster. **(F)** UMAP plot of cell annotation results. **(G)** Violin plots of the specific cell marker genes.

To validate the specific cancer cell subtypes in the MSS group, a single-cell validation cohort including 11,220 cells from CRC patients (5555 cells in the MSI group and 5665 cells in the MSS group) was analyzed. The expression profiles of the CRC cells, as well as the correlation between the numbers of features and counts, are visualized in Supplementary Figures S3A, B. After processing with the same method as in the training cohort, the CRC cells were divided into 20 clusters (Figure 3A and Supplementary Figure S3C), and the top three significantly highly expressed genes in each cluster are shown in Figure 3B. The general differences between the MSI and MSS groups are visualized in Supplementary Figure S3D, while there were no significant batch effects among different patients and cell cycles (Supplementary Figures S3E, F). The absolute amounts and percentages of these 20 cell clusters from CRC patients with MSI and MSS status are visualized in Figures 3C, D. It can be observed that Clusters 12, 13, 14 and 16 account for a significantly higher proportion in the MSS group (exceeding 75%), while Cluster 18 accounts for a significantly higher proportion in the MSI group. After cell annotation, 9 cell types were identified according to their specific cell markers (Figures 3E, F), and the marker genes for each cell type were visualized by violin plots (Figure 3G). For these cell clusters with significant differences, Cluster 12 was annotated as endothelial cells, Cluster 13 was annotated as fibroblasts, Cluster 14 was annotated as cancer cells, Cluster 16 was annotated as myeloid cells and Cluster 18 was annotated as CD4 T cells. These results at least further confirmed the existence of a specific cancer cell subpopulation in the MSS group. Moreover, the proportions of the 9 cell types in the MSI and MSS groups are visualized in Supplementary Figures S2D–F.

Next, DEGs between specific cancer cell Cluster 14 and other cancer cell clusters were identified (Supplementary Table S5). By taking the intersection of the DEGs between different cancer cell subtypes of the single-cell training and validation cohorts, 29 single-cell marker genes were identified in the MSS-specific cancer cell subtype (Supplementary Table S6).

### Calculation of the risk scores of CRC samples and immune analysis

**FIGURE 4:**
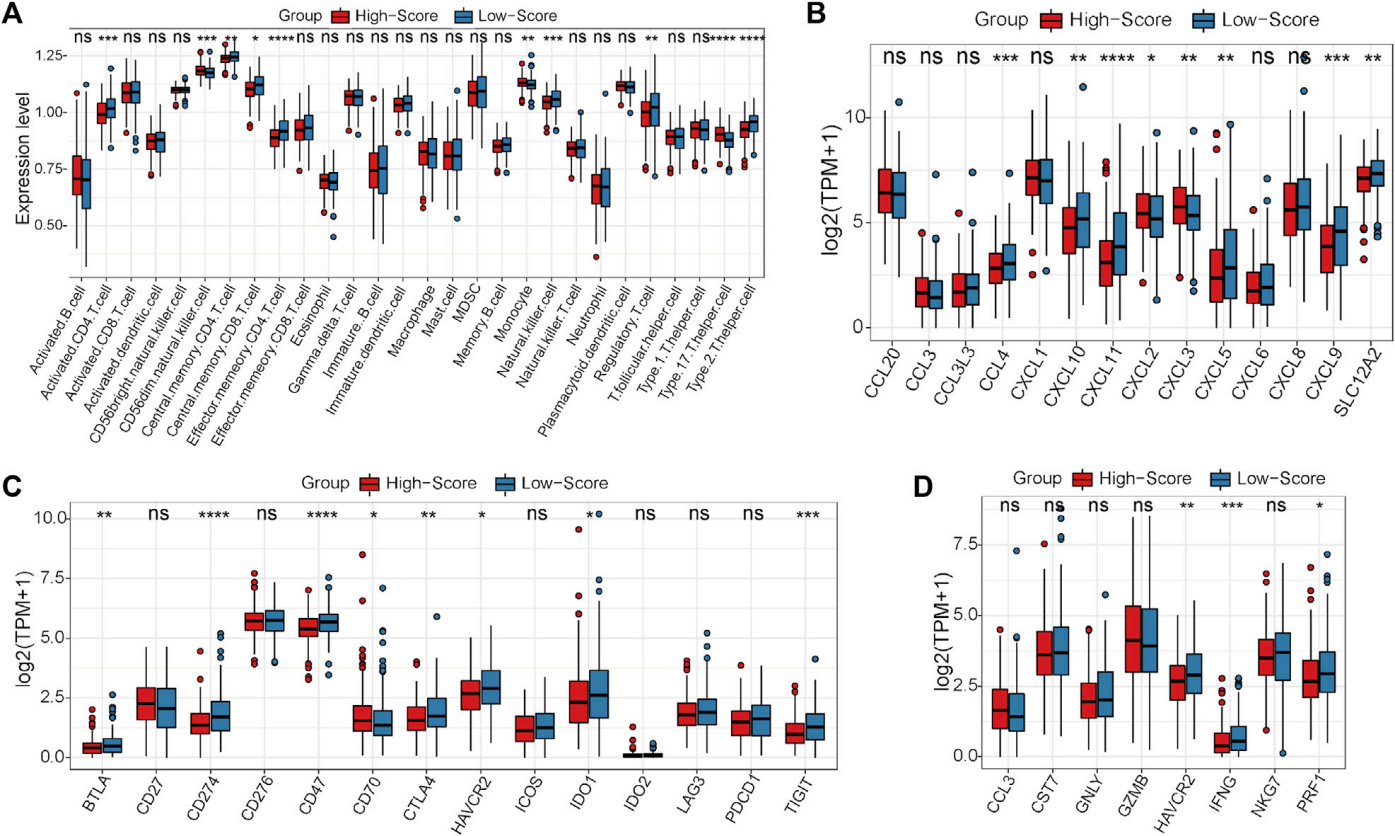
Differential analyses between the high- and low-score groups separated by the MSS-related risk score in the TCGA-CRC cohort. **(A)** The expression levels of the 28 immune cell types in the two groups were visualized by boxplot. **(B–D)** The expression status of classical chemokines **(B)**, immune checkpoint molecules **(C)** and cytotoxic molecules **(D)** between the two risk groups. *p < 0.05, **p < 0.01, ***p < 0.001, ****p < 0.0001, ns, not significant.

First, the expression status of the 29 single-cell marker genes was analyzed in the TCGA-CRC cohort. The results showed that most of these genes were significantly differentially expressed between the MSS and MSI groups (Supplementary Figure S6A). Next, the risk score of each CRC sample with MSS status was calculated by the ssGSEA algorithm based on transcriptome data of these 29 specific single-cell marker genes. Then, the CRC patients were divided into high- and low-score groups according to the median risk score, and most of the single-cell marker genes were also significantly differentially expressed between the two risk groups (Supplementary Figure S6B). The results of survival analysis showed that there was no significant difference in prognosis between the high- and low-score groups (Supplementary Figure S6C). To further investigate the differences in immune characteristics between the two groups, the immune cell abundances of each CRC sample were also calculated by the ssGSEA algorithm based on the expression levels of the corresponding cell marker genes (Supplementary Figure S6D). The differences in immune and clinicopathological characteristics between the high- and low-score groups are visualized in Figure 4A and Supplementary Figure S7. The results showed that most of the significantly differentially expressed immune cells were highly expressed in the low-score group (Figure 4A). Moreover, it is noteworthy that several classical inhibitory immune cells were significantly enriched in the low-score group, such as regulatory T cells (Tregs) and type 2 T helper cells (Th2 cells), while classical inflammatory immune cells were enriched in the high-score group, such as type 17 T helper cells (Th17 cells). These results suggest that CRC patients with MSS status are not sensitive to immunotherapy, which may be caused by the inhibitory immune microenvironment (low-score group) or lack of immune cells (high-score group). To address this hypothesis further, the expression status of the classical chemokines, immune checkpoint molecules and cytotoxic molecules between the two risk groups was analyzed(Park et al., 2022; Propper and Balkwill, 2022; Josan and Neilan, 2023). The results showed that most of these molecules were significantly highly expressed in the low-score group (Figures 4B–D). Taken together, these results indicated that CRC patients with MSS status could be separated into two subtypes according to the MSS-related risk score, and the CRC patients in the low-score group were potentially sensitive to immunotherapy because of their characteristics of “hot” tumors but inhibitory immune microenvironments.

**FIGURE 5:**
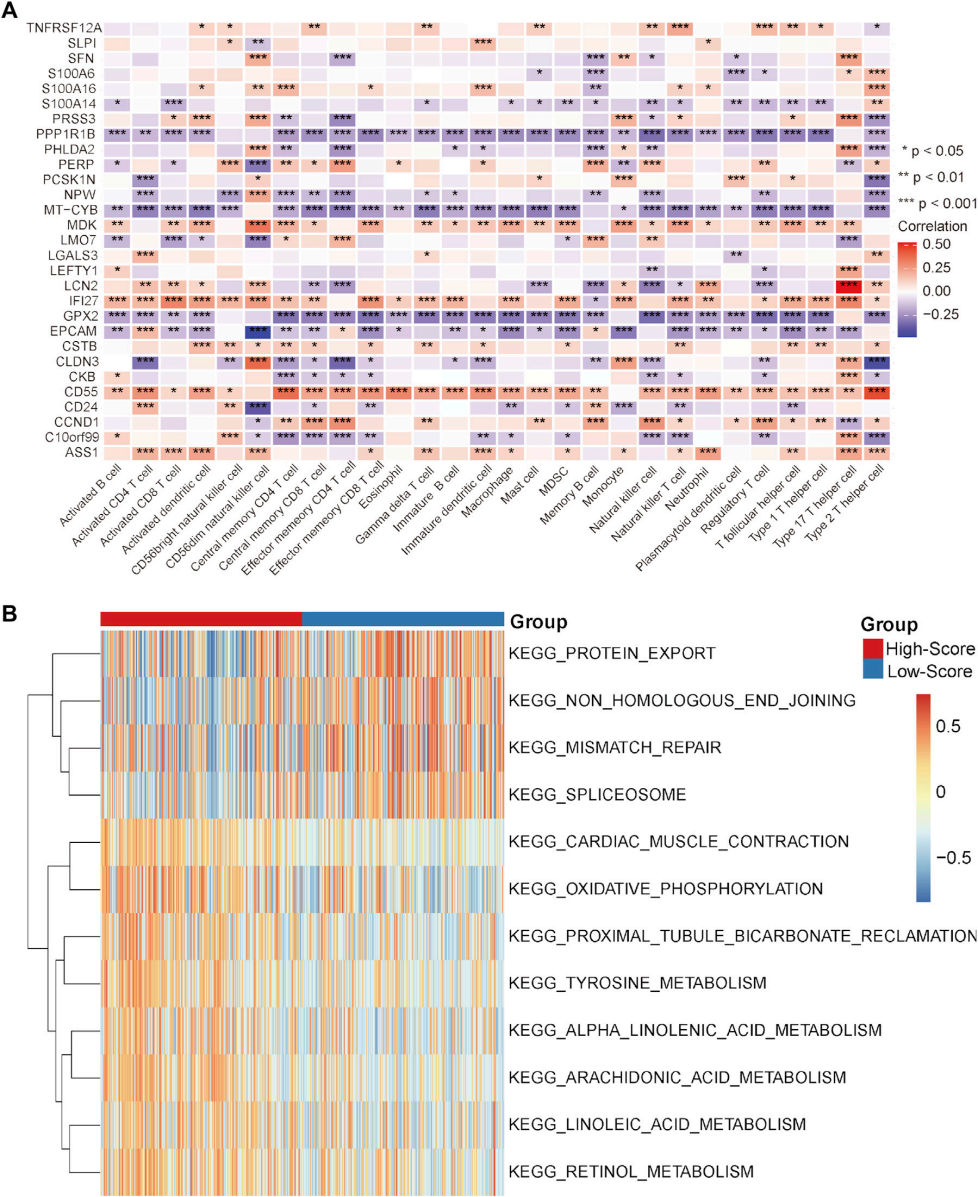
Correlation analysis and GSVA in the TCGA-CRC cohort. **(A)** Correlation heatmap between immune cells and single-cell marker genes. **(B)** GSVA heatmap between the high- and low-score groups. *p < 0.05, **p < 0.01, ***p < 0.001, ****p < 0.0001, ns, not significant.

**FIGURE 6:**
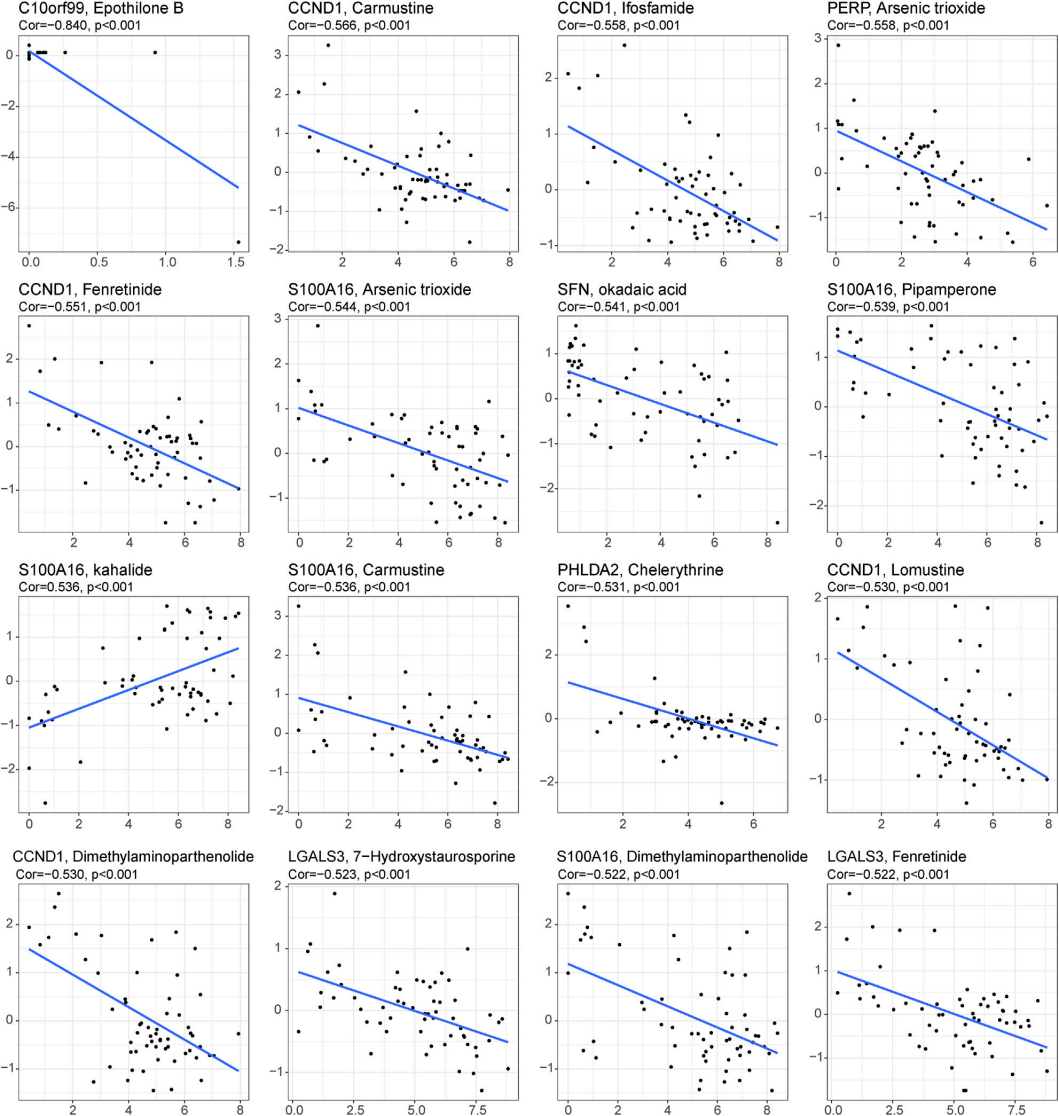
Drug prediction for the 29 single-cell marker genes. Sixteen representative scatter plots of the relationship between drug sensitivity and the expression levels of the 29 genes.

Under this condition, how to reverse the inhibitory immune microenvironments of the CRC patients in the low-score groups becomes the most pressing question. The correlations between the immune cells and single-cell marker genes were then analyzed. The results showed that these genes have a strong correlation with immune cells, especially CD55 with Th2 cells and LCN2 with Th17 cells (Figure 5A). These results indicated that these single-cell marker genes might be potential target genes to regulate the immune microenvironments of CRC patients with MSS status to become synergistic targets to enhance sensitivity to immunotherapy. To search for potential therapeutic drugs targeting these genes, drug sensitivity analyses were conducted by CellMiner. As a result, a total of 956 potential effective drug-gene pairs were identified (Figure 6 and Supplementary Table S7). In addition, to investigate functional differences between the high- and low-risk groups, GSVA was conducted. The results preliminarily revealed that the high-score group was enriched in metabolism-related processes, such as oxidative phosphorylation and tyrosine metabolism, while the low-score group was enriched in genomic stability, such as mismatch repair and non-homologous end joining (Figure 5B).

### External validation of the characteristic differences between the high- and low-score groups in the GEO-CRC cohort

**FIGURE 7:**
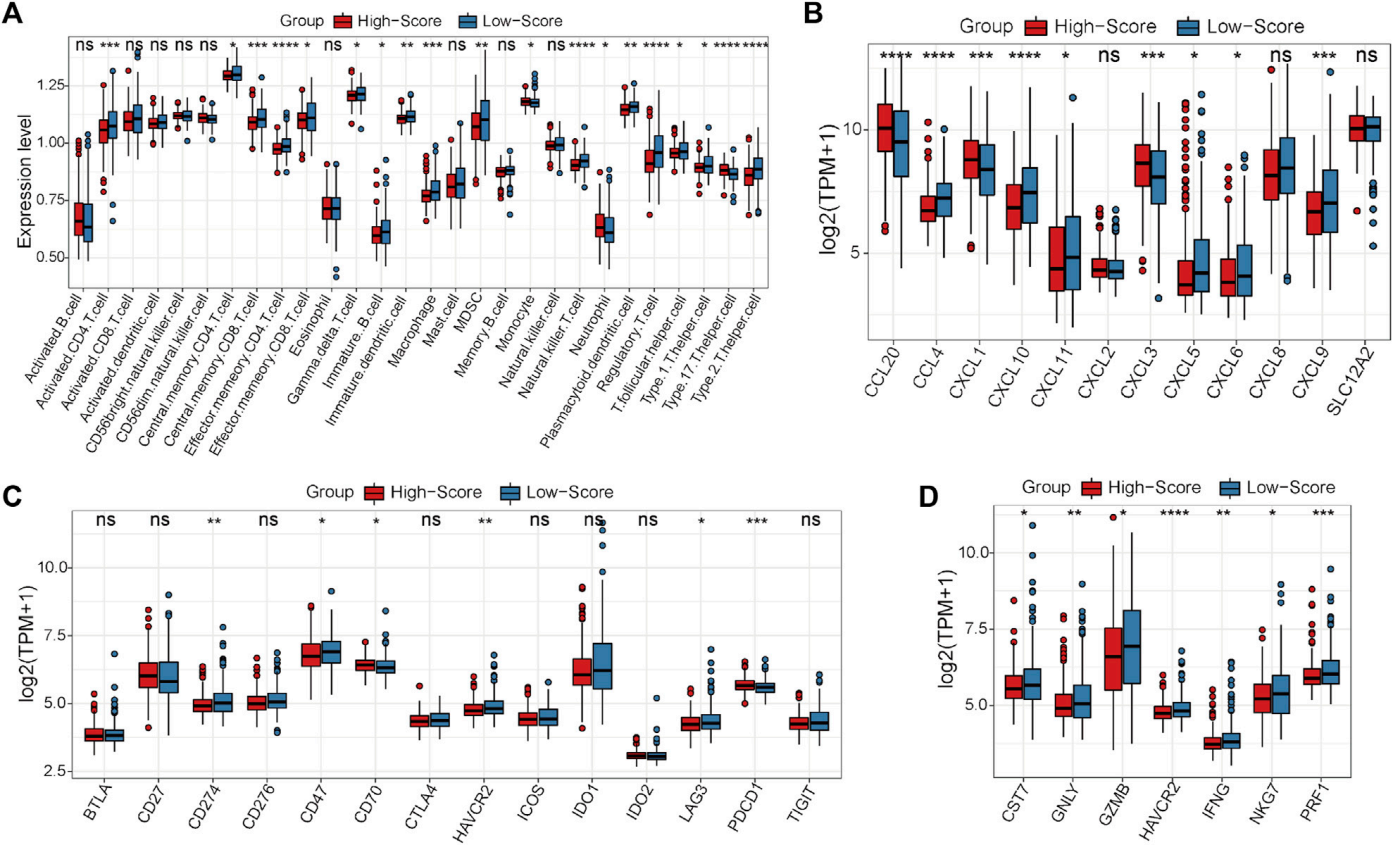
Differential analyses between the high- and low-score groups separated by the MSS-related risk score in the GEO-CRC cohort. **(A)** The expression levels of the 28 immune cell types in the two groups were visualized by boxplot. **(B–D)** The expression status of classical chemokines **(B)**, immune checkpoint molecules **(C)** and cytotoxic molecules **(D)** between the two risk groups. *p < 0.05, **p < 0.01, ***p < 0.001, ****p < 0.0001, ns, not significant.

**FIGURE 8:**
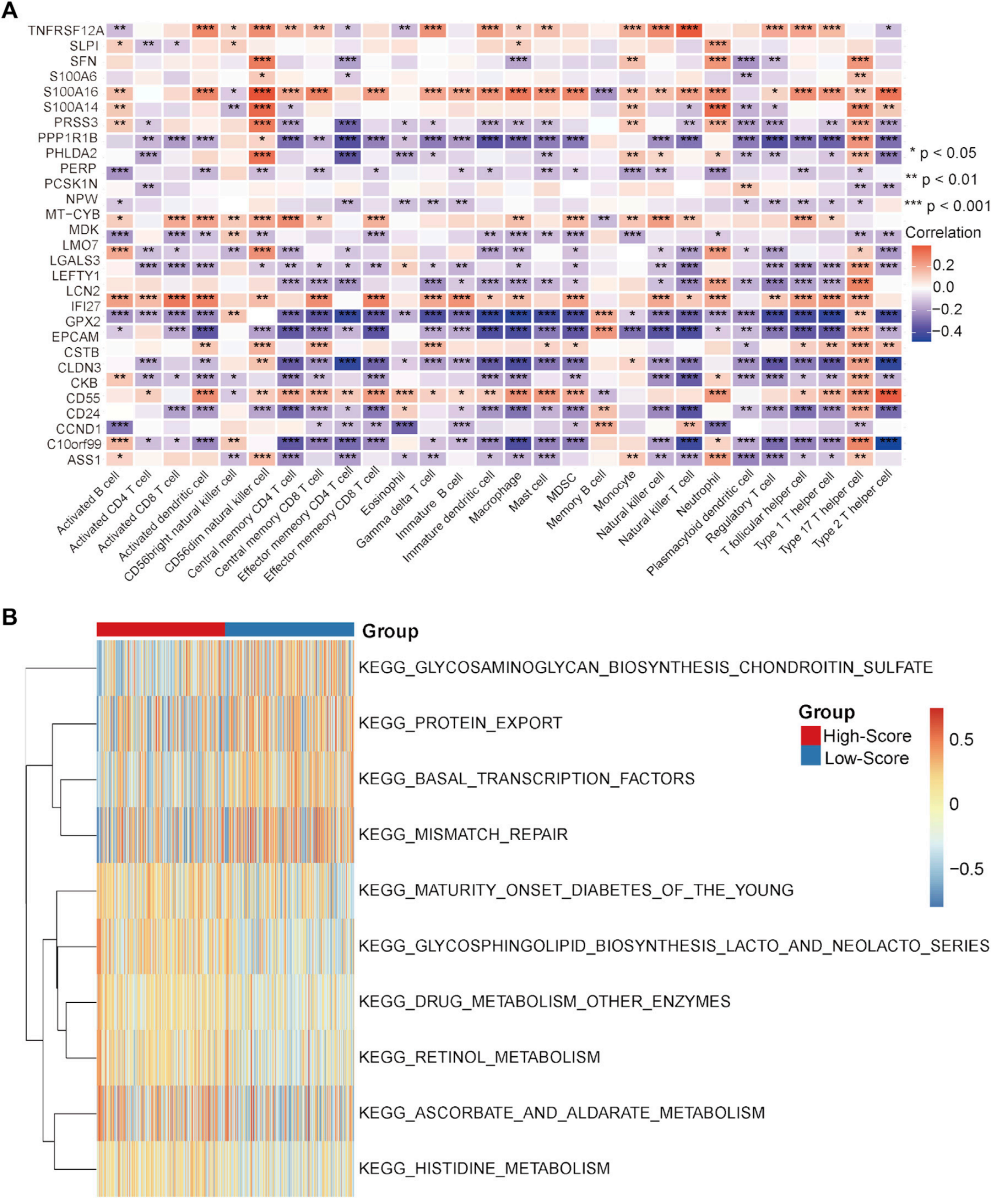
Correlation analysis and GSVA in the GEO-CRC cohort. **(A)** Correlation heatmap between immune cells and single-cell marker genes. **(B)** GSVA heatmap between the high- and low-score groups. *p < 0.05, **p < 0.01, ***p < 0.001, ****p < 0.0001, ns, not significant.

In the GEO-CRC cohort, these 29 single-cell marker genes were also significantly differentially expressed between the dMMR and pMMR groups (Supplementary Figure S8A). The risk scores of the CRC samples with pMMR status were calculated and grouped as in the TCGA-CRC cohort. Most of the single-cell marker genes were significantly differentially expressed between the high- and low-score groups (Supplementary Figure S8B). The results of survival analysis showed that the CRC patients in the low-score group had a significantly worse prognosis (Supplementary Figure S8C). Next, the immune cell abundances of CRC samples were also calculated by the ssGSEA algorithm (Supplementary Figure S8D). The differences in immune and clinicopathological characteristics between the high- and low-score groups are visualized in Figure 7A and Supplementary Figure S9. These results further validated that several classical inhibitory immune cells represented by Tregs, Th2 cells and myeloid-derived suppressor cells (MDSCs) were significantly enriched in the low-score group, while the classical inflammatory immune Th17 cells were enriched in the high-score group. In addition, most of the classical chemokines, immune checkpoint molecules and cytotoxic molecules were also significantly highly expressed in the low-score group (Figures 7B–D). Furthermore, correlation analysis results between the immune cells and single-cell marker genes revealed that these genes have a strong correlation with immune cells (Figure 8A). The GSVA results also confirmed that the high-score group was enriched in metabolism-related processes and that the low-score group was enriched in genomic stability (Figure 8B). Taken together, these results further strongly confirmed the analysis results of the TCGA-CRC cohort that pMMR MSS CRC patients could be separated into two subtypes, and the patients in the low-score group had “hot” tumors with inhibitory immune characteristics, which are potentially more sensitive to immunotherapy. These single-cell marker genes are potential synergistic targets to enhance the sensitivity of CRC patients to immunotherapy.

## Discussion

Immunotherapy represented by ICIs has been approved as the second-line treatment for metastatic CRC with dMMR MSI status, but only a few CRC patients with pMMR MSS status can benefit from immunotherapy due to the lack of immune infiltration and low TMB status(Ganesh et al., 2019; Marcus et al., 2019; Johnson et al., 2022; Weng et al., 2022). Heterogeneities between dMMR MSI and pMMR MSS CRC patients have been preliminarily investigated at the single-cell level, and they have mainly focused on immune cell status(Bao et al., 2020). In this study, we comprehensively dissected the differences between MSI and MSS CRC patients by constructing a single-cell atlas. It is noteworthy that the proportion composition of cancer cell subpopulations was significantly different between the MSI and MSS groups. Moreover, the interactions among different cell types were significantly strengthened in the MSS group compared with the MSI group, and the outgoing signals of the cancer cells were significantly enhanced. These results emphasized the potential core roles of specific cancer cell subtypes in CRC patients with MSS status. Under this condition, the cancer cell subpopulation with a significantly higher proportion in the MSS group was defined as an MSS-specific cancer cell cluster. To elucidate the characteristics of these MSS-specific cancer cells, DEGs between MSS-specific cancer cells and other cancer cell subpopulations were identified. As a result, 29 MSS-specific single-cell marker genes were screened out.

Some studies have aimed to divide pMMR MSS CRC patients into different subgroups to screen potential patients who are sensitive to immunotherapy or a combination therapy scheme(Liang et al., 2021; Gao et al., 2022; Wang et al., 2022). However, these studies have not systematically elucidated the differences between pMMR MSS and dMMR MSI CRC patients at the single-cell level. Moreover, there is no corresponding risk score signature based on differential molecules at the single-cell level to classify CRC patients, so as to determine which patients may be potentially effective for immunotherapy. Under this condition, an MSS-related risk score system was constructed in this study based on the expression levels of the 29 specific single-cell marker genes. This risk score could efficiently divide pMMR MSS CRC patients into two subgroups with significantly different immune characteristics. The high-score group could be identified as the “cold” or “immune-desert” subtype, which is characterized by less immune cell infiltration and low expression of chemokines, immune checkpoint molecules and cytotoxic molecules. In contrast, the low-score group could be identified as the “hot” or “immune-exhausted” subtype, which is characterized by a high abundance of immune cell infiltration and high expression levels of chemokines, cytotoxic molecules and CD8 T cell exhaustion markers, such as CTLA4 and CD274. Moreover, classical inhibitory immune cells (Tregs and Th2 cells) were also enriched in the low-score group. High expression of immune checkpoint molecules is an important predictor of sensitivity to immunotherapy, and it has been reported that reversing the inhibitory immune microenvironment is helpful for enhancing the efficacy of sensitization immunotherapy(Gurjao et al., 2019; Sen et al., 2019; Hosein et al., 2022; Long et al., 2022). Based on this knowledge, these results indicated that the high-score group of pMMR MSS CRC patients could be absolutely insensitive to immunotherapy, while the low-score group CRC patients were the potentially sensitive to immunotherapy if synergistic targets could be identified to reverse their inhibitory immune microenvironment.

In addition, it is noteworthy that some of these 29 specific single-cell marker genes have been studied in tumor immunotherapy(Macor et al., 2015; Strati et al., 2017; Shen et al., 2021; Ahmed et al., 2022; Min et al., 2022), but their role in the response of CRC to immunotherapy is still unclear. Combined with the results of CellMiner, it is suggested that these genes may be potential synergistic targets for enhancing the sensitivity of immunotherapy in CRC patients.

There are still some limitations of the present study. First, whether this MSS-related risk score can efficiently divide pMMR MSS CRC patients into “cold” or “hot” subtypes needs to be verified in a large number of samples from multiple centers. Second, whether the targeted intervention of these 29 single-cell marker genes can reverse the inhibitory immune microenvironment of “hot” CRC patients and thus enhance their sensitivity to immunotherapy needs to be confirmed by basic and clinical studies.

## Conclusion

Taken together, the heterogeneities between the dMMR MSI and pMMR MSS CRC patients were systematically elucidated at the single-cell level for the first time. As a result, a specific MSS cancer cell cluster and a specific MSS cancer cell gene list was identified. Next, an MSS-related risk score was constructed based on the 29 specific single-cell marker genes. This risk score model is useful to categorize CRC samples into “cold” or “hot” tumors, which will help us to judge the potential sensitivity of pMMR MSS CRC patients to immunotherapy. Moreover, this model will also provide fundamental knowledge to screen synergistic targets to sensitize pMMR MSS CRC patients to immunotherapy and improve the overall prognosis of CRC patients.

## Data Availability

The original contributions presented in the study are included in the article/Supplementary Material, further inquiries can be directed to the corresponding author.
