# Supplementary material for: Identification of the molecular characteristics associated with microsatellite status of colorectal cancer patients for the clinical application of immunotherapy
Source: Front Pharmacol. 2023 Feb 6;14:1083449. doi: 10.3389/fphar.2023.1083449 (PMC9939640; doi:10.3389/fphar.2023.1083449)
Supplement: Supplementary file 1 [file DataSheet2.PDF]

1. Analysis of the single-cell RNA sequencing data

```
rm(list = ls())
library(Seurat)
library(tidyverse)
load("srt_GSE132465.MSS.Rdata")
load("srt_GSE144735.MSS.Rdata")
memory.limit(102400)
srt_list <- list(GSE132465 = srt_GSE132465,
                 GSE144735 = srt_GSE144735)
srt3_all <- merge(x = srt_list[[1]],
                  y = srt_list[[2]])
head(srt3_all@meta.data)
save(srt3_all,file="srt_CRC.orig.Rdata")

rm(list = ls())
library(Seurat)
library(tidyverse)
library(stringr)
load("srt_CRC.orig.Rdata")
srt <- srt3_all
mito.genes <- str_subset(string = rownames(srt),
                         pattern = "MT-")
mito.genes <- c("MT-ND1","MT-ND2","MT-CO1","MT-CO2","MT-ATP8","MT-ATP6","MT-
CO3","MT-ND3","MT-ND4L","MT-ND4","MT-ND5","MT-ND6","MT-CYB" )
mito.genes
srt[["percent.mt"]] <- PercentageFeatureSet(srt,
                                            features = mito.genes)
head(srt[["percent.mt"]] )
VlnPlot(object = srt,
        features = c("nFeature_RNA", "nCount_RNA", "percent.mt"),
        group.by    = "group",
        # group.by    = "seurat_clusters",
        log = T,
        pt.size = 0)
RidgePlot(object = srt,
          features = c("nFeature_RNA", "nCount_RNA", "percent.mt"),
          # log = T,
          ncol = 1,
          group.by    = "group")
save(srt, file = "srt_del_mt.Rda")

rm(list = ls())
load("srt_del_mt.Rda")
```

```
srt_list <- SplitObject(srt,
                        split.by = "GEO_source")
for(i in 1:length(srt_list)){
  srt_list[[i]] <- SCTransform(
    srt_list[[i]],
    variable.features.n = 3000,
    # vars.to.regress = c("percent.mt", "S.Score", "G2M.Score"),
    verbose = FALSE)
}
features <- SelectIntegrationFeatures(object.list = srt_list,
                                      nfeatures = 3000)
srt_list <- PrepSCTIntegration(object.list = srt_list,
                               anchor.features = features)
AnchorSet <- FindIntegrationAnchors(object.list = srt_list,
                                    reference = 1,
                                    normalization.method = "SCT",
                                    anchor.features = features)
memory.limit(502400)
srt <- IntegrateData(anchorset = AnchorSet,
                     normalization.method = "SCT")
DefaultAssay(srt) <- "integrated"
save(srt, file = "srt_SCT_final.Rda")

rm(list = ls())
library(Seurat)
library(tidyverse)
library(stringr)
library(patchwork)
load("srt_SCT_final.Rda")
dim(srt)
srt <- RunPCA(object = srt,
              npcs = 50,
              rev.pca = FALSE,
              weight.by.var = TRUE,
              verbose = TRUE,
              ndims.print = 1:5,
              nfeatures.print = 30,
              reduction.key = "PC_")
ElbowPlot(srt,
          ndims = 50)
p1 <- DimPlot(object = srt,
              reduction = "pca",
              # group.by = "group",
              group.by = "GEO_source",
```

```
                    dims = c(1,2),
                    shuffle = TRUE,
                    label = TRUE,
                    label.size = 4,
                    label.color = "black",
                    label.box = TRUE,
                    sizes.highlight = 1,
                    raster=FALSE
                    )
p1
p2 <- DimPlot(object = srt,
                    reduction = "pca",
                    group.by = "Phase",
                    dims = c(1,2),
                    shuffle = TRUE,
                    label = TRUE,
                    label.size = 4,
                    label.color = "black",
                    label.box = TRUE,
                    sizes.highlight = 1)
srt <- RunUMAP(srt,
                            dims = 1:35)
p5 <- DimPlot(object = srt,
                    reduction = "umap",
                    group.by = "group",
                    # group.by = "GEO_source",
                    dims = c(1,2),
                    shuffle = TRUE,
                    label = TRUE,
                    label.size = 4,
                    label.color = "black",
                    label.box = TRUE,
                    sizes.highlight = 1,
                    raster=FALSE)
p5
p6 <- DimPlot(object = srt,
                    reduction = "umap",
                    group.by = "Phase",
                    dims = c(1,2),
                    shuffle = TRUE,
                    label = TRUE,
                    label.size = 4,
                    label.color = "black",
                    label.box = TRUE,
```

```
                        sizes.highlight = 1)
p6
DimPlot(object = srt,
              reduction = "umap",
              group.by = "GEO_source",
              dims = c(1,2),
              shuffle = TRUE,
              label = TRUE,
              label.size = 4,
              label.color = "black",
              label.box = TRUE,
              sizes.highlight = 1)
srt <- FindNeighbors(srt,
                                             k.param = 20,
                                             dims = 1:35)
srt <- FindClusters(srt,
                                           resolution = 0.5,
                                           method = "igraph",
                                           algorithm = 1,
                                           random.seed = 2021)
table(srt@meta.data$seurat_clusters)
DimPlot(object = srt,
              group.by = "seurat_clusters",
              reduction = "pca",
              label = TRUE)
UMAPPlot(object = srt,
                group.by = "seurat_clusters",
                split.by = "group",
                pt.size = 0.5,
                label = TRUE) +
   NoLegend()
UMAPPlot(object = srt,
                group.by = "seurat_clusters",
                pt.size = 0.5,
                label = TRUE)
UMAPPlot(object = srt,
                group.by = "sample",
                pt.size = 0.5,
                label = TRUE)
save(srt, file = "srt_cluster.Rda")

rm(list = ls())
library(tidyverse)
library(reshape2)
```

```
library(patchwork)
library(Seurat)
library(SingleR)
library(celldex)
library(BiocParallel)
library(cowplot)
load(file = "srt_cluster.Rda")
cell_marker <- read.csv("cellmarker.csv")
cell_marker <- cell_marker %>%
  separate_rows(Cell.Marker, sep = ", ") %>%
  na.omit() %>%
  distinct() %>%
  arrange(Cell.Type)
cell_marker$Cell.Marp2ker <- str_trim(cell_marker$Cell.Marker, "left")
p1 <- DimPlot(srt,
              reduction = "umap", # pca, umap, tsne
              group.by    = "seurat_clusters",
              label = T)
p2 <- DotPlot(srt,
              assay = "SCT",
              features = unique(cell_marker$Cell.Marker)) +
  theme(axis.text = element_text(size = 8,
                                 angle = 90,
                                 hjust = 1))
p1 + p2
DotPlot(srt,
        assay = "SCT",
        features = unique(cell_marker$Cell.Marker))+
         # coord_flip()+
  theme_bw()+
  theme(panel.grid = element_blank(),
        axis.text.x=element_text(angle=90,hjust = 1,vjust=0.5))+
  scale_color_gradientn(values = seq(0,1,0.2),colours =
c('#330066','#336699','#66CC66','#FFCC33'))+
  labs(x=NULL,y=NULL)+guides(size=guide_legend(order=3))
Cell_type <- c("0" = "Myeloid cells",
               "1" = "CD8 T cells",
               "2" = "Other T cells",
               "3" = "Cancer cells",
               "4" = "Cancer cells",
               "5" = "CD4 T cells",
               "6" = "Fibroblasts",
               "7" = "Cancer cells",
               "8" = "B cells",
```

```
                    "9" = "Myeloid cells",
                    "10" = "B cells",
                    "11" = "CD4 T cells",
                    "12" = "Endothelial cells",
                    "13" = "CD8 T cells",
                    "14" = "Fibroblasts",
                    "15" = "Cancer cells",
                    "16" = "Fibroblasts",
                    "17" = "Cancer cells",
                    "18" = "Cancer cells",
                    "19" = "Mast cells",
                    "20" = "Myeloid cells")
srt[["cell_type"]] <- unname(Cell_type[srt@meta.data$seurat_clusters])
DimPlot(srt,
                   reduction = "umap",
                   group.by = "cell_type",
                   split.by = "group",
                   label = TRUE,
                   pt.size = 0.5 )
               + NoLegend()
DimPlot(srt,
           reduction = "umap",
           group.by = "cell_type",
           # split.by = "group",
           label = TRUE,
           pt.size = 0.5
           + NoLegend())
VlnPlot(srt,
           assay = "SCT",
           features = c("CD4"),
           group.by = "cell_type",
           pt.size=0)    + NoLegend()
library(MySeuratWrappers)
library(ggplot2)
markers <- cell_marker$Cell.Marker
VlnPlot(srt,
           assay = "SCT",
           group.by    = "cell_type",
           features = markers,
           stacked=T,pt.size=0,
           cols = my36colors,
           direction = "horizontal",
           x.lab = '', y.lab = ''
           )+
```

```
  theme(axis.text.x = element_blank(),
        axis.ticks.x = element_blank())
memory.limit(102400)
all.markers <- FindAllMarkers(srt, only.pos = TRUE, min.pct = 0.1, logfc.threshold = 0.5)
save(all.markers, file = "all.markers.Rdata")
marker_gene <- all.markers[all.markers$cluster %in% c(4,15),]
marker_gene <- marker_gene$gene
save(marker_gene, file = "marker_gene.Rdata")
load("all.markers.Rdata")
Cell_type <- c("0" = "Myeloid cells",
               "1" = "CD8 T cells",
               "2" = "Other T cells",
               "3" = "Cancer cells",
               "4" = "Cancer cells",
               "5" = "CD4 T cells",
               "6" = "Fibroblasts",
               "7" = "Cancer cells",
               "8" = "B cells",
               "9" = "Myeloid cells",
               "10" = "B cells",
               "11" = "CD4 T cells",
               "12" = "Endothelial cells",
               "13" = "CD8 T cells",
               "14" = "Fibroblasts",
               "15" = "Cancer cells",
               "16" = "Fibroblasts",
               "17" = "Cancer cells",
               "18" = "Cancer cells",
               "19" = "Mast cells",
               "20" = "Myeloid cells")
all.markers$cell_type <- unname(Cell_type[all.markers$cluster])
top3 <- all.markers %>%
  group_by(cluster) %>%
  # group_by(cell_type) %>%
  top_n(n = 3, wt = avg_log2FC)
pdf(file = "DoHeatmap_type.pdf",15,14)
DoHeatmap(srt,features = top3$gene,
          group.by = "cell_type"
         # group.by = "seurat_clusters" ,
         #                              group.colors                              =
c("#C77CFF","#7CAE00","#00BFC4","#F8766D","#AB82FF","#90EE90","#00CD00","#00
8B8B","#FFA500"))+
        # scale_fill_gradientn(ccolors = c("navy","white","firebrick3")
         ) + NoLegend()
```

```
dev.off()

plot.data <- srt@meta.data[c("sample", "group","seurat_clusters", "cell_type")]
head(plot.data)
plot.data2 <- plot.data %>%
  group_by(group, (plot.data)) %>%
  dplyr::summarise(num = n())
head(plot.data2)
library(ggsci)
library(plyr)
library(dplyr)
ggplot(plot.data2, aes(x = seurat_clusters,
                       weight = num,
                       fill = group)) +
  geom_bar(position = "dodge") +
  scale_fill_lancet() +
  theme_bw() +
  guides(fill = "none")
ggplot(plot.data2, aes(x = seurat_clusters,
                       weight = num,
                       fill = group)) +
  geom_bar(position = "stack") +
  scale_fill_lancet() +
  theme_bw() +
  ylab("Cell Number") +
  xlab("cell_type")
plot.data3 <- plot.data2 %>%
  ddply("seurat_clusters", transform, percent = num/sum(num) * 100) %>%
  arrange(seurat_clusters)
head(plot.data3)
levels <- unique(plot.data3$seurat_clusters)
plot.data3$seurat_clusters <- factor(plot.data3$seurat_clusters, levels = levels)

ggplot(plot.data3, aes(x = seurat_clusters,
                       weight = percent,
                       fill = group)) +
  geom_bar(position = "stack") +
  scale_fill_lancet() +
  theme_bw() +
  ylab("Cell Percent") +
  xlab("seurat_clusters")

table(srt[["cell_type"]])
Cancer_cells <- subset(srt, cell_type == "Cancer cells")
```

```
Idents(Cancer_cells)
Cancer_cells[["group2"]]        <-        ifelse(Cancer_cells@meta.data$seurat_clusters        ==
c(4,15),"MSS_specific","MSS_non_specific")
Idents(Cancer_cells) <- Cancer_cells@meta.data$group2
Idents(Cancer_cells)

Cancer_cells.diff <- FindMarkers(Cancer_cells,
                                 ident.1 = "MSS_non_specific",
                                 ident.2 = "MSS_specific",
                                 min.pct = 0.5)
Cancer_cells.diff1 <- FindMarkers(Cancer_cells,
                                  ident.1 = "MSS_non_specific",
                                  ident.2 = "MSS_specific",
                                  min.pct = 0.5,
                                  logfc.threshold = log(2),
)
gene <- rownames(Cancer_cells.diff)
save(gene,    file = "gene_scrna.Rdata")
save(Cancer_cells.diff,file = "Cancer_cells.diff.Rdata")

MSS_data <- subset(srt, group == "MSS")
MSI_data <- subset(srt, group == "MSI")
save(MSS_data,file = "MSS_data.Rda")
save(MSI_data,file = "MSI_data.Rda")

rm(list = ls())
library(CellChat)
library(patchwork)
library(Seurat)
load("MSI_data.Rda")    # load("MSS_data.Rda")
AT.str <- GetAssayData(object = MSI_data,
                       assay = "SCT",
                       slot = "counts")
AT.meta.data <- MSI_data@meta.data
AT.cellchat <- createCellChat(object = AT.str,
                              meta = AT.meta.data,
                              group.by = "cell_type")
levels(AT.cellchat@idents)
CellChatDB <- CellChatDB.human
showDatabaseCategory(CellChatDB)
CellChatDB.use <- subsetDB(CellChatDB,
                           search = "Secreted Signaling")
AT.cellchat@DB <- CellChatDB.use
future::plan("multicore", workers = 8)
```

```
AT.cellchat <- subsetData(AT.cellchat)
AT.cellchat <- identifyOverExpressedGenes(AT.cellchat)
AT.cellchat <- identifyOverExpressedInteractions(AT.cellchat)
AT.cellchat <- projectData(AT.cellchat, PPI.human)
AT.cellchat <- computeCommunProb(AT.cellchat, raw.use = TRUE)
AT.cellchat <- filterCommunication(AT.cellchat, min.cells = 10)
df.net <- subsetCommunication(AT.cellchat, slot.name = "netP")
AT.cellchat <- computeCommunProbPathway(AT.cellchat)
AT.cellchat <- aggregateNet(AT.cellchat)
groupSize <- as.numeric(table(AT.cellchat@idents))
netVisual_circle(AT.cellchat@net$count,
                 vertex.weight = groupSize,
                 weight.scale = T,
                 label.edge= F,
                 title.name = "Number of interactions")
netVisual_circle(AT.cellchat@net$weight,
                 vertex.weight = groupSize,
                 weight.scale = T,
                 label.edge= F,
                 title.name = "Interaction weights/strength")
netVisual_heatmap(AT.cellchat,
                  # signaling = "CCL",
                  color.heatmap = "Reds")
netVisual_bubble(AT.cellchat,
                 sources.use = 1:9,
                 targets.use = c(1:9),
                 remove.isolate = FALSE)
plotGeneExpression(AT.cellchat, signaling = "CCL")
AT.cellchat <- netAnalysis_computeCentrality(AT.cellchat,
                                             slot.name = "netP")
ht1 <- netAnalysis_signalingRole_heatmap(AT.cellchat, pattern = "outgoing")
ht2 <- netAnalysis_signalingRole_heatmap(AT.cellchat, pattern = "incoming")
ht1 + ht2
save(AT.cellchat, file = "AT.cellchat_MSI.Rda")
```

2. Analysis of the bulk-sequencing data

```
rm(list = ls())
library(tidyverse)
clinic_data <-
read.table("TCGA.COADREAD.sampleMap_COADREAD_clinicalMatrix", header = T, sep
= "\t")
clinic_data   <- clinic_data[,c(1,4)]
```

```
clinic_data <- clinic_data[clinic_data$CDE_ID_3226963 %in% c("MSI-H","MSI-L","MSS"),]
table(clinic_data$CDE_ID_3226963)
names(clinic_data) <- c("Sample","MS_Status")
clin <- data.table::fread ("TCGA_CRC.tsv")
sample <- intersect(clin$Sample, clinic_data$Sample)
clin <- clin[clin$Sample %in% sample,]
clinic_data <- clinic_data[clinic_data$Sample %in% sample,]
clin <- merge(clin, clinic_data, by="Sample")
clin2 <- clin[,c(1,14,15)]
clin2 <- clin2 %>% na.omit()
clin <- clin [clin$Sample %in% clin2$Sample,]
save(clin, file = "clin.Rdata")

rm(list = ls())
library(tidyverse)
load("clin.Rdata")
load("CRC_tpm.Rdata")
load("gene_cluster.Rdata")
sample <- intersect(colnames(exp),clin$Sample)
exp <- exp[,sample]
clin <- clin[clin$Sample %in% sample,]
table(clin$MS_Status)
group2 <- ifelse(clin$MS_Status %in% "MSS","MSS","MSI")
table(group2)
clin$group <- group2
clin_MSI <- clin[clin$group %in% "MSI",]
clin_MSS <- clin[clin$group %in% "MSS",]
sample_MSI <- clin_MSI$Sample
sample_MSS <- clin_MSS$Sample
exp <- exp[aaa,]
exp <- exp %>% t() %>% as.data.frame()
group <- ifelse(rownames(exp) %in% sample_MSI,"MSI","MSS")
exp$group <- group
table(group)
exp_barplot <- exp %>% gather(key = aaa,value = Proportion,1:29)
library(ggpubr)
library(RColorBrewer)
mypalette <- colorRampPalette(brewer.pal(8,"Set1"))
pdf(file = "1.diff_exp_2.pdf",8,4)
ggplot(exp_barplot,aes(aaa,Proportion,fill = group)) +
  geom_boxplot(outlier.shape = 21,color = "black") +
  theme_bw() +
  labs(x = " ", y = "log2(TPM+1)") +
```

```
  theme(legend.position = "top") +
  theme(axis.text.x = element_text(angle=60,hjust =1))+
  scale_fill_manual(values = mypalette(21)[c(1,4)])+
  stat_compare_means(aes(group = group,label = ..p.signif..),
                     method = "t.test")
dev.off()

clin <- clin[clin$MS_Status %in% "MSS",]
save(clin, file = "clin_MSS.Rdata")

rm(list = ls())
library(tidyverse)
load("CRC_tpm.Rdata")
load("clin_MSS.Rdata")
sample <- intersect(colnames(exp),clin$Sample)
exp <- exp[,sample]
clin <- clin[clin$Sample %in% sample,]
save(exp,clin,file = "analysis_use.Rdata")

rm(list = ls())
library(tidyverse)
library(GSVA)
load("analysis_use.Rdata")
exp <- exp %>% as.matrix()
cell_set <- readxl::read_xlsx("ssGSEA_marker2.xlsx")
a <- table(cell_set$Score) %>% as.data.frame()
gene_extract <- function(x) {
  x$Metagene %>% unique() %>% as.character()
}
cell_set <- lapply(split(cell_set,cell_set$Score),gene_extract)
Cell_gsva <- gsva(exp,cell_set, method = "ssgsea")
Cell_gsva <- Cell_gsva %>% t() %>% as.data.frame()
Cell_gsva <- Cell_gsva %>% rownames_to_column(var = "Sample")
range(Cell_gsva$Cancer_cell_score)
clin2 <- clin[,c(1,14,15)]
Cell_gsva <- merge(Cell_gsva, clin2, by = "Sample")
Cell_gsva <- Cell_gsva %>% column_to_rownames(var = "Sample")
Cell_gsva$Status <- ifelse(Cell_gsva$Status == "0:LIVING",0,1)
library(tidyverse)
library(survMisc)
library(survminer)
exp <- Cell_gsva
exp$signature_by2 <- ifelse(exp$Cancer_cell_score > median(exp$Cancer_cell_score),'High-
Score','Low-Score')
```

```
table(exp$signature_by2)
exp_high <- exp[(exp$signature_by2)=="High-Score",]
exp_low <- exp[(exp$signature_by2)=="Low-Score",]
ID_high <- rownames(exp_high)
ID_low <- rownames(exp_low)
save(ID_high,ID_low, file = "group_ID_2.Rdata")

exp2 <- exp[,2:4]
fit <- survfit(Surv(OS, Status)~signature_by2,data = exp2)

p <- ggsurvplot(fit, conf.int=F, pval=T, risk.table=T, legend.labs = c('High-Score','Low-Score'),
                legend.title='Risk Score',
                risk.table.height = 0.3)
dir_name <- 'Results_Plot'
pdf(paste0('./',dir_name,'/Plot2_Signature_KMplot.pdf'),onefile = F)
print(p)
dev.off()

rm(list = ls())
library(tidyverse)
load("analysis_use.Rdata")
library(GSVA)
exp <- exp %>% as.matrix()
cell_set <- readxl::read_xlsx("ssGSEA_marker.xlsx")
a <- table(cell_set$`Cell type`) %>% as.data.frame()
gene_extract <- function(x) {
  x$Metagene %>% unique() %>% as.character()
}
cell_set <- lapply(split(cell_set,cell_set$`Cell type`),gene_extract)
Cell_gsva <- gsva(exp,cell_set, method = "ssgsea")
Cell_gsva <- Cell_gsva %>% t() %>% as.data.frame()
Cell_gsva <- Cell_gsva %>% rownames_to_column(var = "Sample")
save(Cell_gsva,file = "Cell_gsva.Rdata")
load("Cell_gsva.Rdata")
exp <- Cell_gsva
colnames(exp) <- gsub(pattern = " ",replacement = ".", colnames(exp))
exp2 <- exp %>% column_to_rownames(var = "Sample") %>% t() %>% as.data.frame()
bk <- c(seq(0,0.2,by = 0.01),seq(0.21,0.85,by=0.01))
library(pheatmap)
library(RColorBrewer)
load("group_ID_2.Rdata")
clin2 <- clin[,c(1,3,4,5,6,12,16,18,19,20,22,23)]
group <- ifelse(colnames(exp2) %in% ID_high,"High-Score","Low-Score")
table(group)
```

```r
annotation_col = data.frame(
   group =    group)
rownames(annotation_col) = colnames(exp2)
annotation_col <- annotation_col %>% rownames_to_column(var = "Sample")
annotation_col <- merge(annotation_col, clin2, by="Sample")
annotation_col <- annotation_col %>% column_to_rownames(var = "Sample")
exp_h <- exp2[,ID_high]
exp_l <- exp2[,ID_low]
exp_h <- exp_h %>% rownames_to_column(var = "cell")
exp_l <- exp_l %>% rownames_to_column(var = "cell")
exp2 <- merge(exp_h,exp_l,by="cell")
exp2 <- exp2 %>% column_to_rownames(var = "cell")
library(RColorBrewer)
mypalette <- colorRampPalette(brewer.pal(8,"Set1"))
ann_colors = list(     group = c("High-Score" = mypalette(22)[c(1)], "Low-Score" = mypalette(22)[c(4)]))
pdf(file = "3.heatmap-110.pdf",10,8)
pheatmap(
   exp2,
   annotation_col = annotation_col,
   annotation_colors = ann_colors,
   breaks = bk,
   cluster_cols = F,
   scale = "row",
   cluster_row = T,
   border_color = NA,
   show_colnames = F,
   show_rownames = T,
)
dev.off()
exp_barplot <- exp %>%
   gather(key = Cell_type,value = Proportion,2:29)
pdf(file = "4.immune_barplot.pdf",8,4)
ggplot(exp_barplot,aes(Cell_type,Proportion,fill = Cell_type)) +
   geom_boxplot(outlier.shape = 21,coulour = "black") + theme_bw() +
   labs(x = "Cell_Type", y = "Expression level") +
   theme(axis.text.x = element_blank()) + theme(axis.ticks.x = element_blank()) +
   scale_fill_manual(values = mypalette(28))
dev.off()
exp$group <- group
exp_barplot <- exp %>%
   gather(key = Cell_type,value = Proportion,2:29)
library(ggpubr)
pdf(file = "5.data_barplot_2.pdf",8,5)
```

```
ggplot(exp_barplot,aes(Cell_type,Proportion,fill = group)) +
  geom_boxplot(outlier.shape = 21,color = "black") +
  theme_bw() +
  labs(x = "Cell Type", y = "Expression level") +
  theme(legend.position = "top") +
  theme(axis.text.x = element_text(angle=60,hjust =1))+
  scale_fill_manual(values = mypalette(22)[c(1,4)])+
  stat_compare_means(aes(group = group,label = ..p.signif..),
                     method = "wilcox.test")
dev.off()

rm(list=ls())
library(dplyr)
library(tidyr)
library(ggplot2)
library(tidyverse)
load("Cell_gsva.Rdata")
load("analysis_use.Rdata")
load("gene_cluster.Rdata")
immune <- Cell_gsva
immune <- immune %>% column_to_rownames(var = "Sample")
exp <- exp [aaa,]
sample <- intersect(colnames(exp),rownames(immune))
immune <- immune [sample,]
exp <- exp[,rownames(immune)]
identical(rownames(immune), colnames(exp))
gene <- rownames(exp)
immuscore <- function(gene){
  y <- as.numeric(exp[gene,])
  colnames <- colnames(immune)
  do.call(rbind,lapply(colnames, function(x){
    dd   <- cor.test(as.numeric(immune[,x]),y,type="spearman")
    data.frame(gene=gene,immune_cells=x,cor=dd$estimate,p.value=dd$p.value )
  }))
}
data <- do.call(rbind,lapply(gene,immuscore))
head(data)
data$pstar <- ifelse(data$p.value < 0.05,
                     ifelse(data$p.value < 0.01,
                            ifelse(data$p.value < 0.001,"***", "**"), "*"), "")
pdf(file = "6.gene_immune_cor.pdf",12,7)
ggplot(data, aes(immune_cells, gene)) +
  geom_tile(aes(fill = cor), colour = "white",size=1) +
  scale_fill_gradient2(low = "blue",mid = "white",high = "red") +
```

```
  geom_text(aes(label=pstar),col ="black",size = 5) +
  theme_minimal() +
  theme(axis.title.x=element_blank(),
        axis.ticks.x=element_blank(),
        axis.title.y=element_blank(),
        axis.text.x = element_text(angle = 45, hjust = 1),
        axis.text.y = element_text(size = 8))+
  labs(fill =paste0(" * p < 0.05","\n\n",
                    "** p < 0.01","\n\n",
                    "*** p < 0.001","\n\n",
                    "Correlation"))
dev.off()

rm(list = ls())
library(tidyverse)
load("analysis_use.Rdata")
exp <- exp %>% t() %>% as.data.frame()
load("group_ID_2.Rdata")
exp <- exp [c(ID_high,ID_low),]
exp_immune                                                                     <-
exp[,c("PDCD1","CD274","CD276","BTLA","CTLA4","LAG3","HAVCR2","TIGIT","ICOS
","IDO1","IDO2","CD47","CD70","CD27")]
group <- if_else(rownames(exp_immune) %in% ID_high,"High-Score","Low-Score")
table(group)
exp_immune$group <- group
table(exp_immune$group)
library(RColorBrewer)
mypalette <- colorRampPalette(brewer.pal(8,"Set1"))
exp_barplot <- exp_immune %>%
  gather(key = immune_gene,value = Proportion,1:14)
library(ggpubr)
pdf(file = "7.immune_cell_exp.pdf",8,4)
ggplot(exp_barplot,aes(immune_gene,Proportion,fill = group)) +
  geom_boxplot(outlier.shape = 21,color = "black") +
  theme_bw() +
  labs(x = " ", y = "log2(TPM+1)") +
  theme(legend.position = "top") +
  theme(axis.text.x = element_text(angle=60,hjust =1))+
  scale_fill_manual(values = mypalette(21)[c(1,4)])+
  stat_compare_means(aes(group = group,label = ..p.signif..),
                     method = "kruskal.test")
dev.off()

rm(list=ls())
```

```r
library(GSEABase)
library(GSVA)
library(tidyverse)
load("analysis_use.Rdata")
exp <- exp %>% as.matrix()
geneset <- getGmt("c2.cp.kegg.v7.2.symbols.gmt")
KEGG_gsva<- gsva(exp,
                 geneset,
                 mx.diff=FALSE,
                 verbose=T,
                 parallel.sz=1)
save(KEGG_gsva,file = "CRC_KEGG_gsva.Rdata")
load("CRC_KEGG_gsva.Rdata")
library(limma)
load("group_ID_2.Rdata")
KEGG_gsva1 <-KEGG_gsva[,ID_high]
KEGG_gsva2 <-KEGG_gsva[,ID_low]
KEGG_gsva1 <- KEGG_gsva1 %>% as.data.frame() %>% rownames_to_column(var =
"gsva_term")
KEGG_gsva2 <- KEGG_gsva2 %>% as.data.frame() %>% rownames_to_column(var =
"gsva_term")
KEGG_gsva <- merge(KEGG_gsva1,KEGG_gsva2,by="gsva_term")
KEGG_gsva <- KEGG_gsva %>% column_to_rownames(var = "gsva_term") %>% as.matrix()
group <- ifelse(colnames(KEGG_gsva) %in% ID_high,"High","Low")
table(group)
design <- model.matrix(~0+factor(group))
colnames(design) <- levels(factor(group))
rownames(design) <- colnames(KEGG_gsva)
contrast_matrix<-makeContrasts("High-Low",
                               levels = design)
fit <- lmFit(KEGG_gsva,design)
fit2 <- contrasts.fit(fit, contrast_matrix)
fit2 <- eBayes(fit2)
res <- decideTests(fit2, p.value=0.05)
summary(res)
de_KEGG <- topTable(fit2,
                    adjust.method="BH",
                    p.value=1, lfc=0)
de_KEGG <- topTable(fit2,number = 186,
                    adjust.method="BH",
                    p.value=1, lfc=0)
library(pheatmap)
de_KEGG <- de_KEGG[de_KEGG$P.Value<0.05 & abs(de_KEGG$logFC) > 0.2,]
dat <- KEGG_gsva[rownames(KEGG_gsva)%in%rownames(de_KEGG),]
```

```
group <-(sort(group))
annotation_col <- data.frame(group)
rownames(annotation_col) <- colnames(dat)
library(RColorBrewer)
mypalette <- colorRampPalette(brewer.pal(8,"Set1"))
ann_colors = list(    group = c(High = mypalette(22)[c(1)], Low = mypalette(22)[c(4)]))
pdf(file = "8.GSVA_heatmap.pdf",10,6)
pheatmap::pheatmap(dat, width = 20, height = 11,
                   annotation_col = annotation_col,
                   show_colnames = F,
                   cluster_cols = F,
                   annotation_colors = ann_colors,)
dev.off()

rm(list = ls())
load("analysis_use.Rdata")
library(tidyverse)
exp <- exp %>% t() %>% as.data.frame()
load("gene_cluster.Rdata")
exp_random <- exp[,aaa]
exp_random <- exp_random %>% rownames_to_column(var = "sample")
load("group_ID_2.Rdata")
exp_random$group <- ifelse(exp_random$sample %in% ID_high,'High-Score','Low-Score')
table(exp_random$group)
library(ggpubr)
library(RColorBrewer)
mypalette <- colorRampPalette(brewer.pal(8,"Set1"))
compaired <- list(c("Low-Score", "High-Score"))

rm(list = ls())
load("analysis_use.Rdata")
library(tidyverse)
exp <- exp %>% t() %>% as.data.frame()
load("gene_cluster.Rdata")
exp_random <- exp[,aaa]
load("group_ID_2.Rdata")
group <- ifelse(rownames(exp_random) %in% ID_high,'High-Score','Low-Score')
exp_random$group <- group
table(exp_random$group)
exp_barplot <- exp_random %>% gather(key = aaa,value = Proportion,1:29)
library(ggpubr)
library(RColorBrewer)
mypalette <- colorRampPalette(brewer.pal(8,"Set1"))
```

```
pdf(file = "9.diff_exp.pdf",8,4)
ggplot(exp_barplot,aes(aaa,Proportion,fill = group)) +
  geom_boxplot(outlier.shape = 21,color = "black") +
  theme_bw() +
  labs(x = " ", y = "log2(TPM+1)") +
  theme(legend.position = "top") +
  theme(axis.text.x = element_text(angle=60,hjust =1))+
  scale_fill_manual(values = mypalette(21)[c(1,4)])+
  stat_compare_means(aes(group = group,label = ..p.signif..),
                     method = "t.test")
dev.off()

rm(list = ls())
load("analysis_use.Rdata")
library(tidyverse)
exp <- exp %>% t() %>% as.data.frame()
gene <- read.csv(file = "1.Cytotoxicity.csv")
gene <- gene$Gene
exp_random <- exp[,gene]
load("group_ID_2.Rdata")
group <- ifelse(rownames(exp_random) %in% ID_high,'High-Score','Low-Score')
exp_random$group <- group
table(exp_random$group)
exp_barplot <- exp_random %>% gather(key = gene,value = Proportion,1:8)
library(ggpubr)
library(RColorBrewer)
mypalette <- colorRampPalette(brewer.pal(8,"Set1"))
pdf(file = "10.Cytotoxicity.pdf",4,4)
ggplot(exp_barplot,aes(gene,Proportion,fill = group)) +
  geom_boxplot(outlier.shape = 21,color = "black") +
  theme_bw() +
  labs(x = " ", y = "log2(TPM+1)") +
  theme(legend.position = "top") +
  theme(axis.text.x = element_text(angle=60,hjust =1))+
  scale_fill_manual(values = mypalette(21)[c(1,4)])+
  stat_compare_means(aes(group = group,label = ..p.signif..),
                     method = "t.test")
dev.off()

rm(list = ls())
load("analysis_use.Rdata")
library(tidyverse)
exp <- exp %>% t() %>% as.data.frame()
```

```
gene <- read.csv(file = "1.Chemokines.csv")
gene <- gene$Gene
exp_random <- exp[,gene]
load("group_ID_2.Rdata")
group <- ifelse(rownames(exp_random) %in% ID_high,'High-Score','Low-Score')
exp_random$group <- group
table(exp_random$group)
exp_barplot <- exp_random %>% gather(key = gene,value = Proportion,1:14)
library(ggpubr)
library(RColorBrewer)
mypalette <- colorRampPalette(brewer.pal(8,"Set1"))
pdf(file = "11.Chemokines.pdf",5,4)
ggplot(exp_barplot,aes(gene,Proportion,fill = group)) +
  geom_boxplot(outlier.shape = 21,color = "black") +
  theme_bw() +
  labs(x = " ", y = "log2(TPM+1)") +
  theme(legend.position = "top") +
  theme(axis.text.x = element_text(angle=60,hjust =1))+
  scale_fill_manual(values = mypalette(21)[c(1,4)])+
  stat_compare_means(aes(group = group,label = ..p.signif..),
                     method = "t.test")
dev.off()
```
