# Supplementary figures and images for: Identification of the molecular characteristics associated with microsatellite status of colorectal cancer patients for the clinical application of immunotherapy
Source: Front Pharmacol. 2023 Feb 6;14:1083449. doi: 10.3389/fphar.2023.1083449 (PMC9939640; doi:10.3389/fphar.2023.1083449)

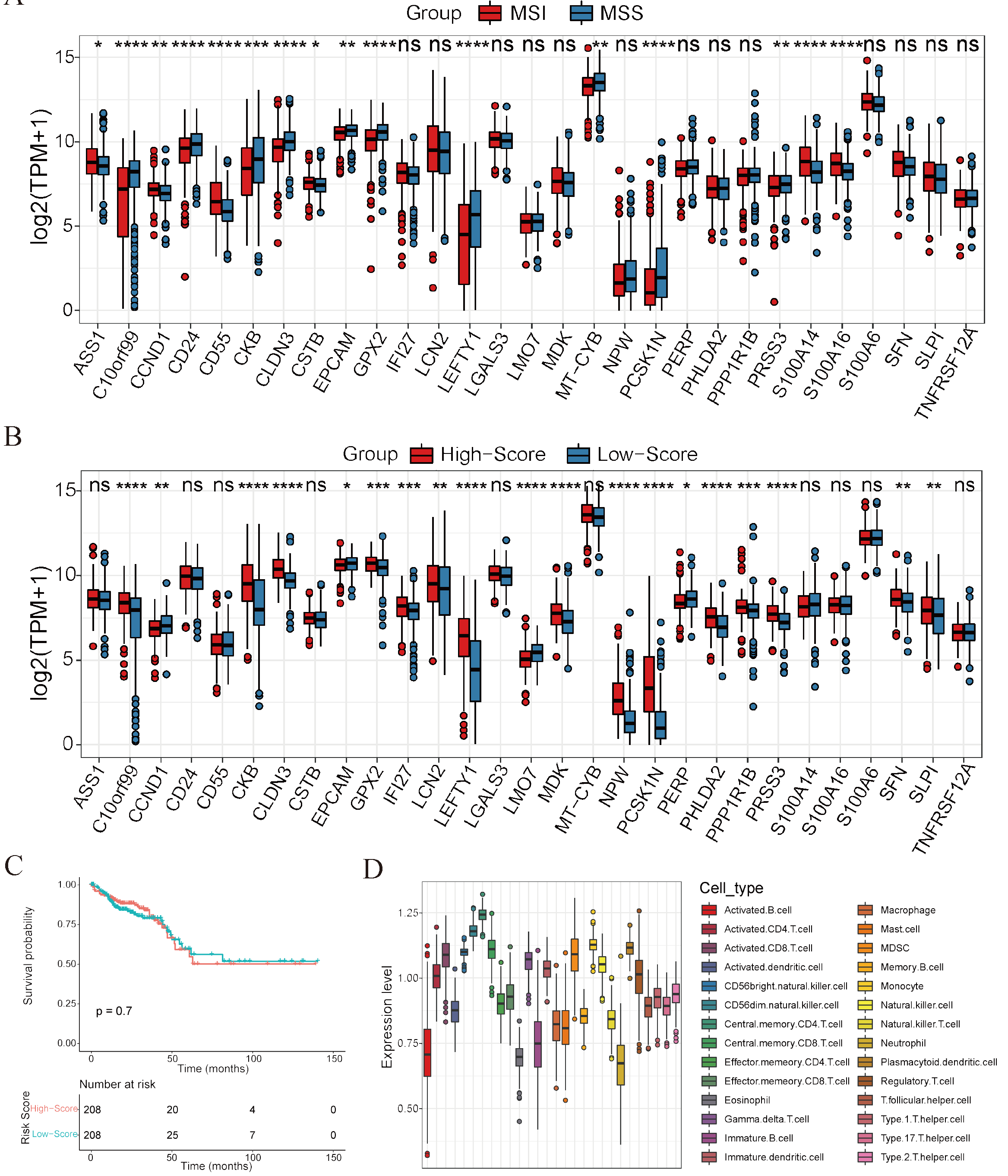

Supplement: Supplementary file 4 [file Image6.TIF]

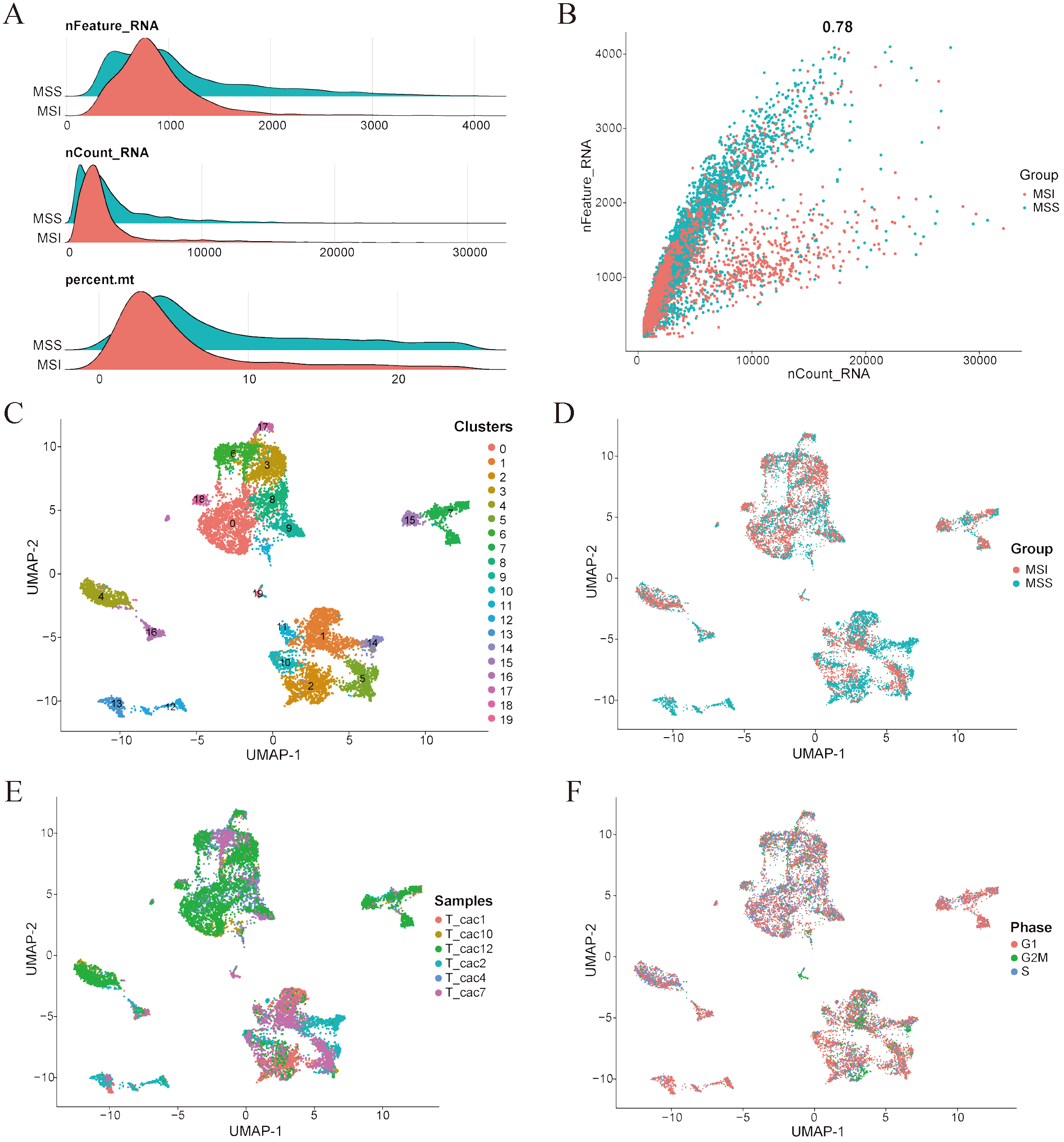

Supplement: Supplementary file 5 [file Image3.TIF]

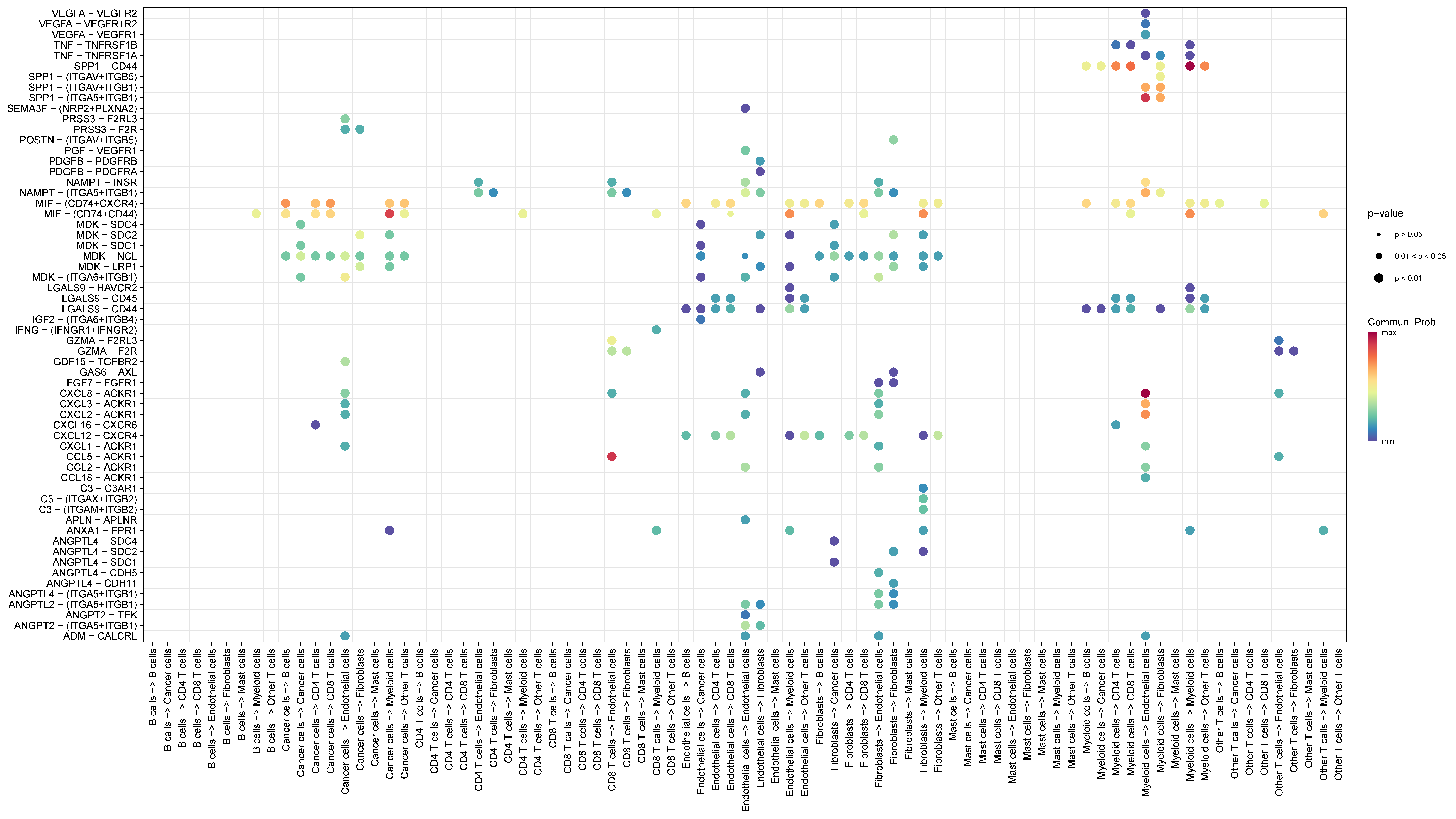

Supplement: Supplementary file 6 [file Image4.TIF]

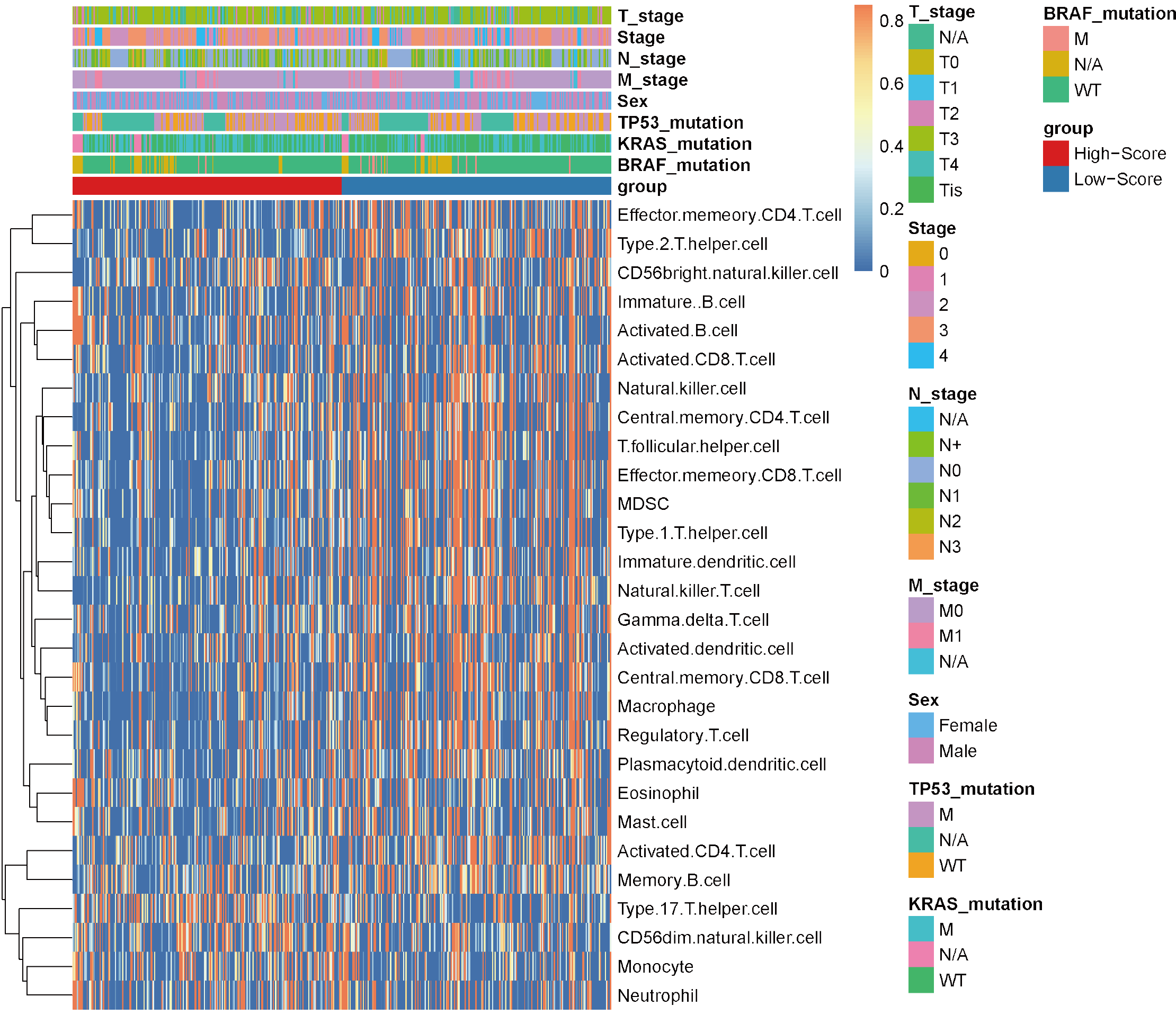

Supplement: Supplementary file 7 [file Image9.TIF]

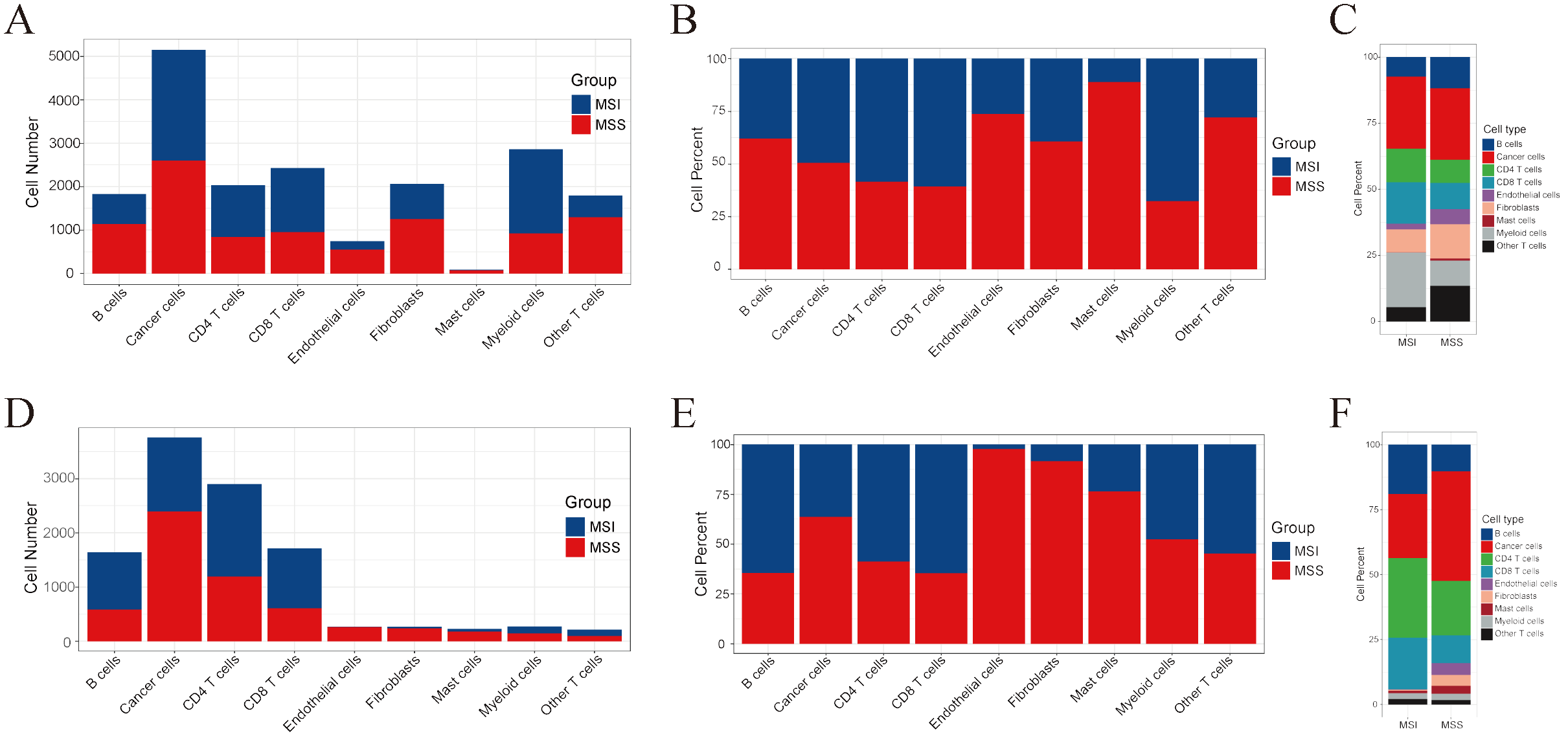

Supplement: Supplementary file 8 [file Image2.TIF]

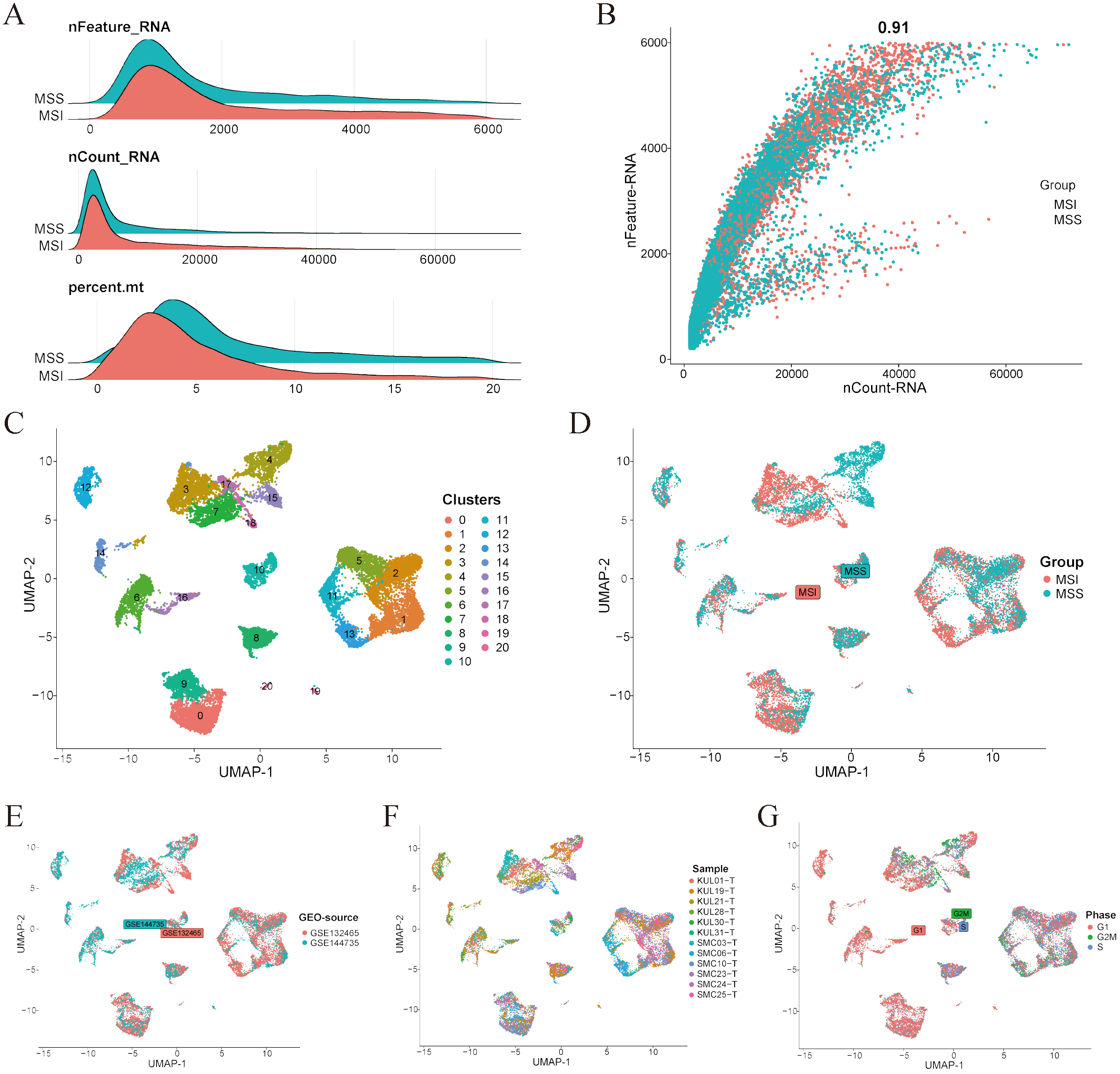

Supplement: Supplementary file 9 [file Image1.TIF]

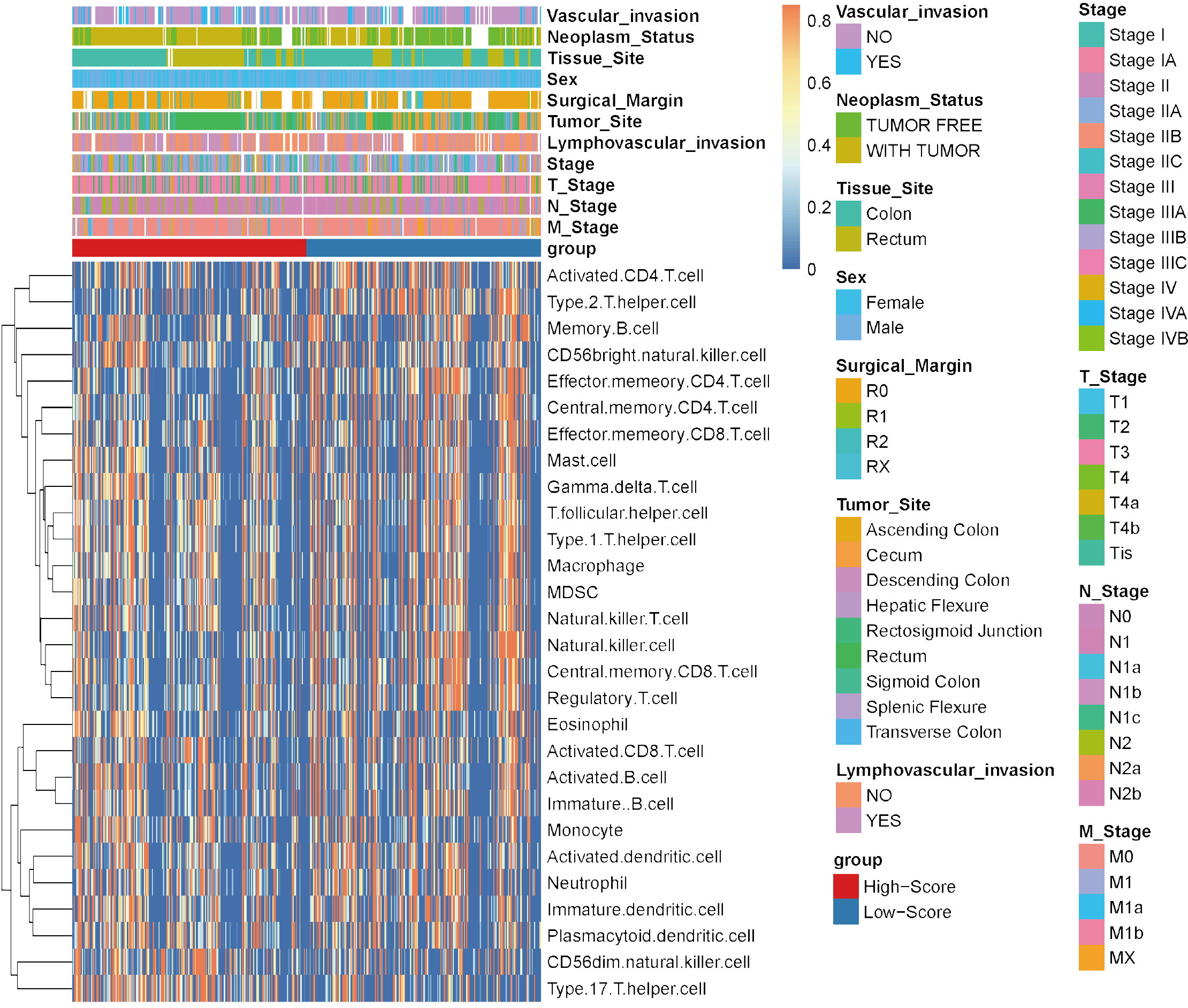

Supplement: Supplementary file 10 [file Image7.TIF]

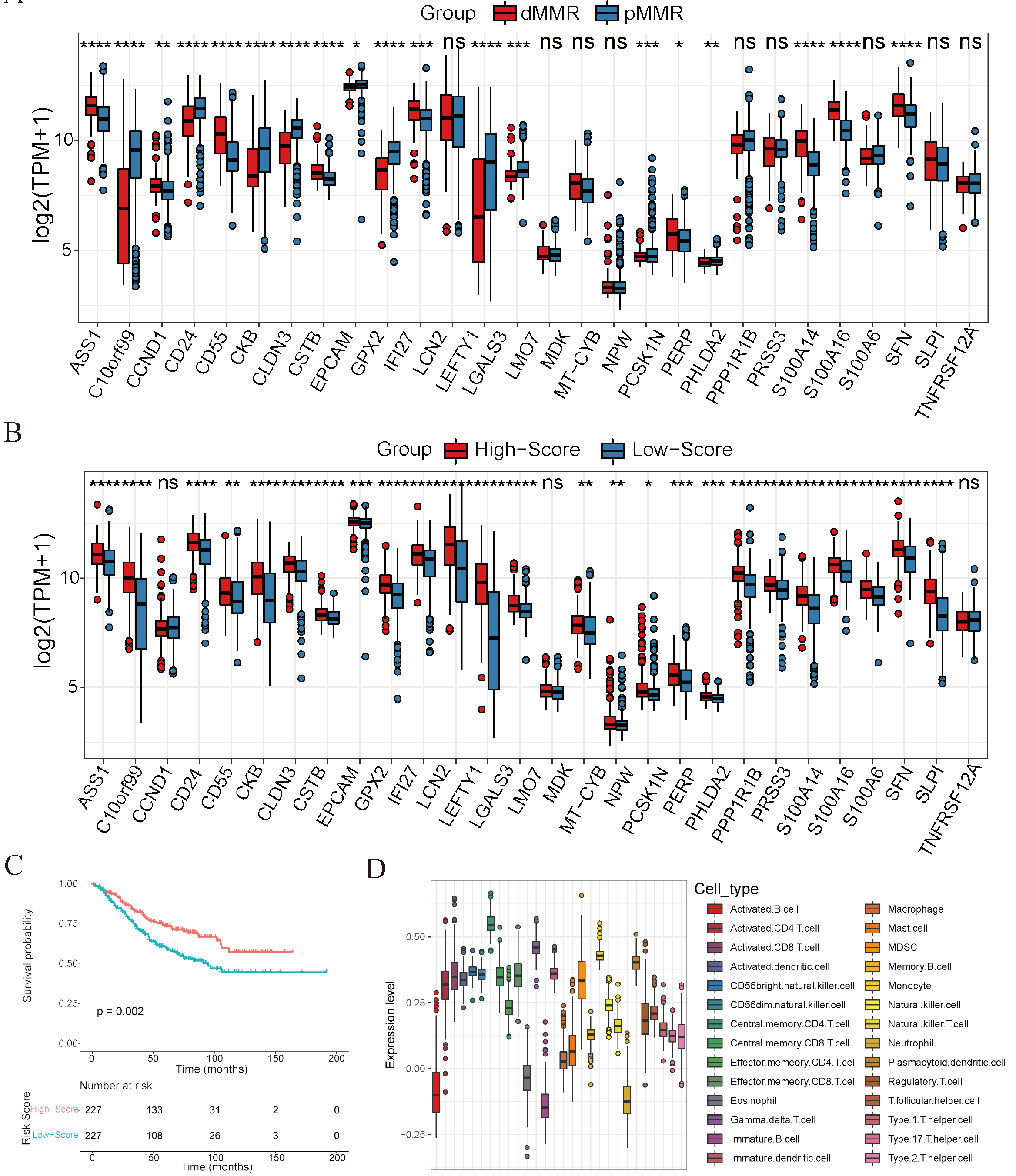

Supplement: Supplementary file 14 [file Image8.TIF]

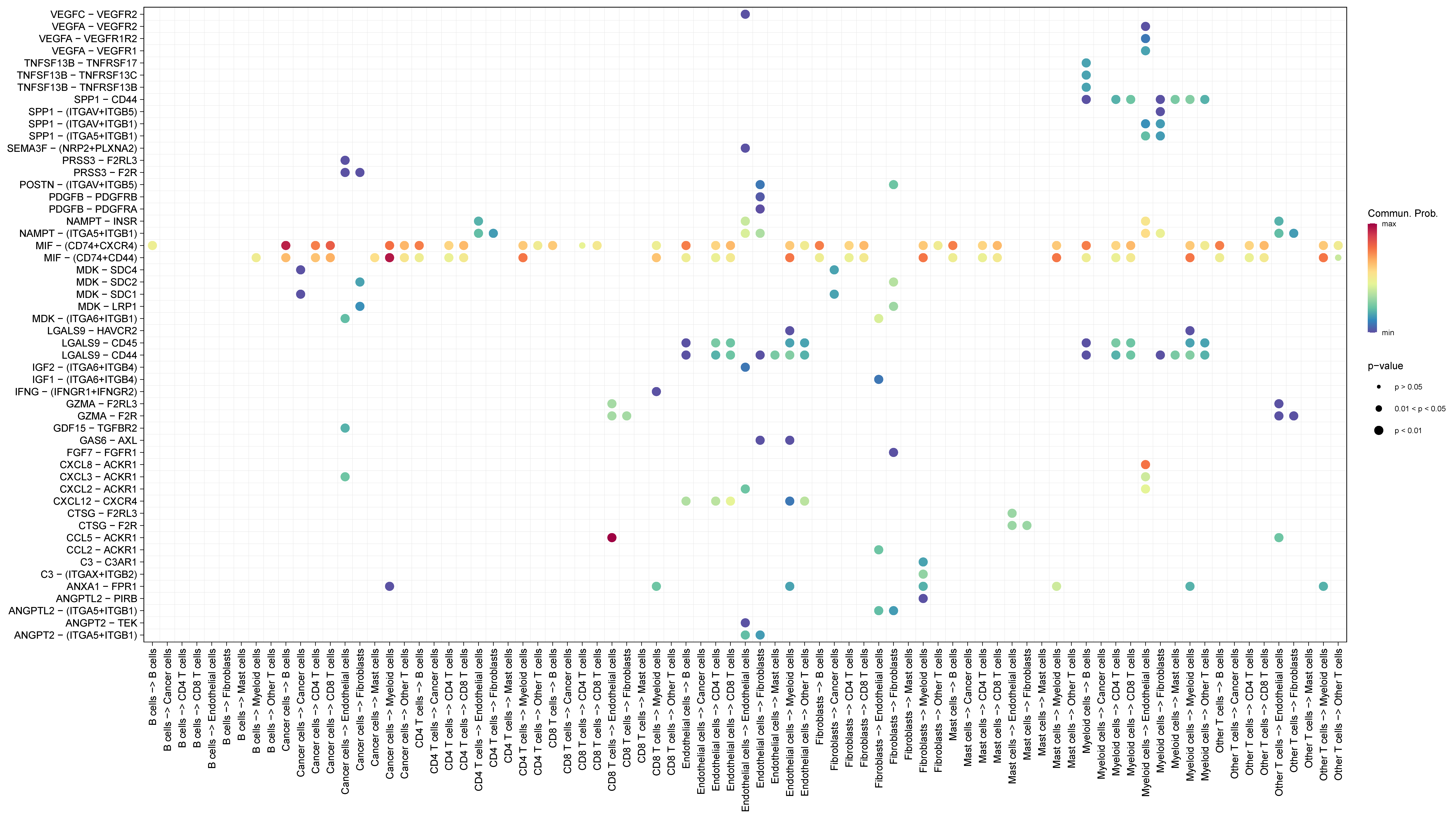

Supplement: Supplementary file 15 [file Image5.TIF]
